# Effects of Microbial Transformation on the Biological Activities of Prenylated Chalcones from *Angelica keiskei*

**DOI:** 10.3390/foods11040543

**Published:** 2022-02-14

**Authors:** Yina Xiao, Ik-Soo Lee

**Affiliations:** College of Pharmacy, Chonnam National University, Gwangju 61186, Korea; yogurtxiao@163.com

**Keywords:** *Angelica keiskei*, microbial transformation, isobavachalcone, 4-hydroxyderricin, xanthoangelol, cytotoxicity, tyrosinase inhibition

## Abstract

Microbial transformation is an alternative method for structural modification. The current study aimed at application of microbial transformation for discovering new derivatives and investigating the structure-activity relationship of isobavachalcone (**1**), 4-hydroxyderricin (**2**), and xanthoangelol (**3**) isolated from the herb *Angelica keiskei*. In the initial screening process, **1**–**3** were incubated with microbes using a two-stage fermentation method and analyzed through TLC monitoring. The screening results showed that *Rhizopus oryzae* and *Mucor hiemalis* were able to transform **1** and **2**, respectively. Additionally, *M. hiemalis* and *Mortierella ramanniana* var. *angulispora* were able to transform **3**. Following scale-up fermentation, four new (**4**, **5**, **7**, and **10**) and five known (**6**, **8**, **9**, **11**, and **12**) metabolites were produced. Cytotoxicity of all the compounds (**1**–**12**) was investigated using three human cancer cell lines including A375P, HT-29, and MCF-7 by MTT method. Meanwhile, the tyrosinase inhibitory activity of **1**–**12** was evaluated using l-tyrosine as a substrate. Overall, **1** and **3** displayed the highest cytotoxicity, and **5** and **7** exhibited the most potent tyrosinase inhibitory activity with relatively low cytotoxicity. This allowed us to postulate that the introduction of 4′-*O*-glucopyranosyl group led to the reduction in cytotoxicity and improvement in tyrosinase inhibitory activity.

## 1. Introduction

*Angelica keiskei* (Umbelliferae) is a perennial leafy herb, mainly distributed in Asian countries, including Korea and Japan. The herb is named as ‘Myeong-il yeob’ in Korean and ‘Ashitaba’ in Japanese, both literally meaning tomorrow’s leaves. Another prevalent name in Korean for *A. keiskei* is ‘Sinsuncho’ meaning the herb of eternal youth [1]. In daily life, it is drunk as a tea and cooked as a vegetable. Traditionally, it is consumed as medicinal herb with tonic, mild cathartic, diuretic, and galactagogue effects [2]. Preceding phytochemical investigations on *A. keiskei* revealed the presence of abundant prenylated chalcones in its leaves, stems, and roots [3]. These chalcones are proposed as effective agents for diverse health-beneficial properties such as anti-tumor [4], anti-inflammatory [5], anti-bacterial [6], anti-diabetic [7], anti-melanogenic [8], and anti-obesity [9] effects. Particularly, 4-hydroxyderricin (HD) and xanthoangelol (XT) are the predominant prenylchalcones in this herb, with relative abundances of 1.97% and 5.05%, respectively [10]. HD and XT have been proposed as potent cytotoxic agents that promote apoptosis and suppress tumor-induced angiogenesis in cancer cells [11]. After surgically removing implanted tumors, the administration of HD or XT inhibited metastasis and increased overall survival in mice. They were considered as promising therapeutic agents for the treatment of melanoma [12,13]. Isobavachalcone (IBC) is another bioactive prenylchalcone isolated from *A. keiskei* [14]. Accumulative studies have demonstrated that IBC suppressed the proliferation and induced cell apoptosis in diverse cancer cell lines including colorectal cancer, liver cancer, breast cancer, leukemia, tongue squamous cell carcinoma [15]. Meanwhile, IBC demonstrated reduced cytotoxicity toward normal cells in comparison with cancer cells. After oral administration in mice, IBC inhibited the growth of subcutaneous HL-60 xenograft tumor without obvious toxicity [16].

Microbial transformation has been used as an effective means for structural modification of natural products at non-activated positions. It has advantages over conventional chemical synthesis largely due to its simpler operation, higher selectivity, and milder conditions [17]. In some cases, biological activities may have been enhanced for the transformed metabolites [18,19]. Previous studies on the microbial transformation of natural bioactive chalcones identified diverse metabolites with promising activities [20,21,22]. The aim of the study was to apply microbial transformation of three major bioactive prenylchalcones (**1**–**3**) in order to identify new derivatives and evaluate their biological activities to understand their structure-activity relationship.

## 2. Materials and Methods

### 2.1. Materials and Reagents

The herbal material *Angelica keiskei* was obtained in September 2020 and identified by the herb company Damaon (Yeongcheon, Gyeongsangbuk-do, Korea). Voucher specimen (AK2009) has been deposited at the Herbarium of the College of Pharmacy, Chonnam National University (Gwangju, Korea).

The microbial media including malt extract, peptone, d-glucose, and potato dextrose were obtained from Becton, Dickinson and Co. (Sparks, MD, USA). Sucrose was obtained from Sigma-Aldrich (St. Louis, MO, USA). Dulbecco’s modified Eagle medium (DMEM), penicillin, and streptomycin were purchased from Gibco (Invitrogen, Carlsbad, CA, USA). Fetal bovine serum (FBS) was purchased from Welgene Inc. (Gyeongsan, Gyeongsangbuk-do, Korea). Phosphate-buffered saline (PBS) tablets were obtained from Takara Korea Biomedical Inc. (Seoul, Korea). 3-(4,5-Dimethylthiazol-2-yl)-2,5-diphenyltetrazolium bromide (MTT) was obtained from Thermo Fisher Scientific (Waltham, MA, USA). Demethylzeylasteral (DZ) used as a reference standard in the bioassay was purchased from Biopurify Phytochemicals, Ltd. (Chengdu, China). Tyrosinase from mushroom and l-tyrosine were purchased from Sigma-Aldrich (St. Louis, MO, USA) and Daejung Chemicals and Metals Co., Ltd. (Siheung, Gyeonggi-do, Korea), respectively. Kojic acid used as a reference standard in the bioassay was purchased from Tokyo Chemical Industry Co., Ltd. (Tokyo, Japan).

The microbial strains including *Cunninghamella elegans* var. *elegans* KCTC 6992, *Mortierella ramanniana* var. *angulispora* KCTC 6137, *Mucor hiemalis* KCTC 26779, *Mucor plumbeus* KCCM 60265, *Rhizopus oryzae* KCCM 60556 were purchased from the Korean Collection for Type Cultures (KCTC, Daejeon, Korea) or Korean Culture Center of Microorganisms (KCCM, Seoul, Korea). The human cancer cell lines A375P, HT-29, and MCF-7 were obtained from the Korean Cell Line Bank (Seoul, Korea).

### 2.2. Isolation and Identification of Chalcones **1**–**3** from the Aerial Parts of A. keiskei

The air-dried herbal material (2.0 kg) was powdered and extracted by soaking in 95% ethanol (10 L × 3). After concentration of the filtrate, the residue (432 g) was suspended in water and sequentially partitioned with dichloromethane (CH_2_Cl_2_) and ethyl acetate (EtOAc). The crude CH_2_Cl_2_ residue (59.0 g) was subsequently separated by silica gel column chromatography using a gradient of *n*-hexane/EtOAc (30:1–1:1) and CH_2_Cl_2_/methanol (10:1–0:1) to yield eleven fractions (Fr. A–K). Fr. D (3.76 g) was further subjected to a Sephadex LH-20 column chromatography using methanol to yield five subfractions D1–D5. Subfr. D5 was separated using high performance liquid chromatography (HPLC) to provide substrates **1** (50 mg), **2** (95 mg), and **3** (110 mg). The structures of **1**–**3** were established as isobavachalcone (**1**) [23], 4-hydroxyderricin (**2**) [24] and xanthoangelol (**3**) [25] by ^1^H- and ^13^C-nuclear magnetic resonance (NMR) spectral analysis (see Supplementary Materials Figures S2–S7).

### 2.3. Preparation and Analysis of Screening Samples for Microbial Transformation

Fermentation experiments were carried out in two kinds of media. The malt medium consisting of peptone (1 g), d-glucose (20 g), and malt extract (20 g) was prepared in 1 L of distilled water for the incubation of *M. hiemalis*. The potato sucrose medium consisting of sucrose (20 g) and potato dextrose (24 g) was prepared in 1 L of distilled water for the culture of *C. elegans* var. *elegans*, *M. ramanniana* var. *angulispora*, *M. plumbeus*, or *R. oryzae*.

**Scheme 1:**
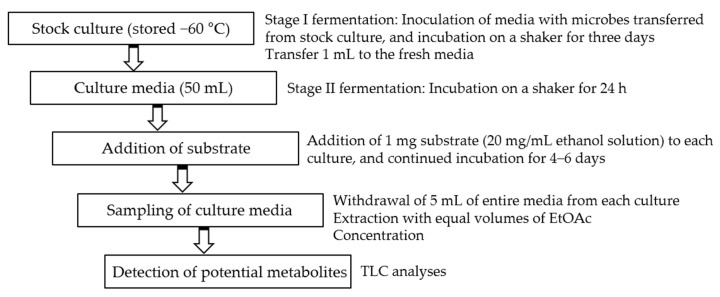
Two-stage fermentation method for screening.

Initial fermentations were performed in 50 mL media. A two-stage fermentation method was employed in all experiments [26]. After inoculation and continued incubation for 24 h, the solution of each substrate (**1**–**3**) in ethanol (20 mg/mL) was distributed to each flask. The concentration of each substrate in the media was 0.02 mg/mL. Incubation was further continued for 4–6 days. The two parallel controls were conducted under the same conditions, i.e., substrate controls (substrate in microorganism-free culture media) and culture controls (microorganisms in substrate-free culture media). Substrate-containing microbial media were sampled by withdrawing 5 mL of entire media at every two days after addition of substrate. They were extracted with equal volumes of EtOAc. The organic layers were concentrated and spotted on thin layer chromatography (TLC) plates, and developed with chloroform/methanol (6:1). After spraying anisaldehyde-sulfuric acid reagent, the TLC plates were heated over 100 °C for detection of potential metabolites (Scheme 1).

On the basis of TLC analyses, the microbes *M. plumbeus* KCCM 60556 and *M. hiemalis* KCTC 26779 were selected for scale-up fermentations of substrates **1** and **2**, respectively, as they showed higher transformational efficiency, while the microbes *M. hiemalis* KCTC 26779 and *Mortierella ramanniana* var. *angulispora* KCTC 6137 were selected for scale-up fermentations of **3**, as they showed potential capability of transforming **3** (See Supplementary Materials Figure S1).

### 2.4. Preparation and Isolation of Scale-Up Samples to Obtain Metabolites **4**–**11**

Scale-up fermentation of each substrate (**1**–**3**) was conducted following the aforementioned procedures in 150 mL of pre-cultured media. The scale-up fermentation culture of each substrate (**1**–**3**) was extracted thrice with equal volume of EtOAc. The combined EtOAc extract was evaporated in vacuo to give a residue.

The organic residue of **1** cultured with *R. oryzae* was separated on HPLC eluted with 53% to 84% methanol to yield metabolites **4** (t_R_ = 30.4 min, 2.8 mg) and **6** (t_R_ = 34.9 min, 6.5 mg) together with fractions RO1 (t_R_ = 34.1 min) and RO2 (t_R_ = 36.8 min) at 2.0 mL/min. Metabolite **5** (t_R_ = 32.0 min, 3.7 mg) was obtained by a further purification of fr. RO1 on HPLC with 55% to 72% methanol. Metabolite **7** (t_R_ = 36.8 min, 3.2 mg) was obtained by a further purification of fr. RO2 on HPLC with a gradient of 53% to 85% methanol.

The organic residue of **2** cultured with *M. hiemalis* was separated on HPLC with 58% to 85% methanol to furnish **8** (t_R_ = 16.6 min, 1.8 mg,) and **9** (t_R_ = 26.4 min, 2.2 mg) at 2.0 mL/min.

The organic residue of **3** cultured with *M. hiemalis* was separated on HPLC with 58% to 97% methanol to yield metabolite **10** (3.5 mg, t_R_ = 32.8 min) at 2.0 mL/min.

The organic residue of **3** cultured with *Mortierella ramanniana* var. *angulispora* was separated on HPLC with 58% to 78% methanol to provide fraction MR1 (t_R_ = 32.8 min). Metabolites **11** (2.4 mg, t_R_ = 14.8 min) and **12** (2.2 mg, t_R_ = 17.8 min) were yielded by a further purification of fr. MR1 on HPLC with *n*-hexane: anhydrous EtOH (85: 15, *v*/*v*) at 1.0 mL/min.

### 2.5. Structural Characterization of New Metabolites **4**, **5**, **7**, and **10**

#### 2.5.1. 3′-(3-Hydroxy-3-methylbutyl)-4′-*O*-β-d-glucopyranosyl-4,2′-dihydroxychalcone (**4**)

Yellow solid; ${\left[\alpha \right]}_{D}^{20}$–25.2 (c 0.10, MeOH); UV (MeOH) λ_max_: 371 nm; IR ν_max_: 3333, 2855, 1632, 1233, 990 cm^−1^; HRESIMS *m/z*: 527.1892 [M+Na]^+^ (calcd. for C_26_H_32_O_10_Na, 527.1893); ^1^H- and ^13^C-NMR spectral data: see Table 1.

#### 2.5.2. 3′-(3-*O*-Methyl-3-methylbutyl)-4′-*O*-β-d-glucopyranosyl-4,2′-dihydroxychalcone (**5**)

Yellow solid; ${\left[\alpha \right]}_{D}^{20}$–26.9 (c 0.10, MeOH); UV (MeOH) λ_max_: 365 nm; IR ν_max_: 3330, 2927, 1780, 1632, 1284, 1105, 1074 cm^−1^; HRESIMS *m/z*: 541.2049 [M+Na]^+^ (calcd. for C_27_H_34_O_10_Na, 541.2050); ^1^H- and ^13^C-NMR spectral data: see Table 1.

#### 2.5.3. 3′-(3-*O*-Ethyl-3-methylbutyl)-4′-*O*-β-d-glucopyranosyl-4,2′-dihydroxychalcone (**7**)

Yellow solid; ${\left[\alpha \right]}_{D}^{20}$–46.5 (c 0.10, MeOH); UV (MeOH) λ_max_: 363 nm; IR ν_max_: 3319, 2919, 1604, 1285, 1105, 835 cm^−1^; HRESIMS *m*/*z*: 555.2208 [M+Na]^+^ (calcd. for C_28_H_36_O_10_Na, 555.2206); ^1^H- and ^13^C-NMR spectral data: see Table 1.

#### 2.5.4. 4′-*O*-β-d-Glucopyranosyl xanthoangelol (**10**)

Yellow solid; ${\left[\alpha \right]}_{D}^{20}$–28.0 (c 0.10, MeOH); UV (MeOH) λ_max_: 365 nm; IR ν_max_: 3342, 2926, 1713, 1631, 1233, 1076 cm^−1^; HRESIMS *m/z*: 577.2414 [M+Na]^+^ (calcd. for C_31_H_38_O_9_Na, 577.2414); ^1^H- and ^13^C-NMR spectral data: see Table 1.

### 2.6. Acid Hydrolysis of **4**, **5**, **7**, and **10**

Each solution of compounds **4**, **5**, **7**, and **10** (each 0.8 mg) in 2 mL 2N HCl was heated at 85 °C for 3 h. After cooling and neutralization, the reaction mixture was extracted with EtOAc. The water layer was concentrated and confirmed by TLC in comparison with authentic d-glucose as a standard. The organic layer containing the aglycone of **10** was determined as the corresponding substrate **3** by comparing their retention time on HPLC.

### 2.7. Cytotoxic Activity Evaluation

The cytotoxicity assays were conducted according to the MTT method as previously described [27] using human melanoma A375P, human colorectal HT-29, and human breast cancer MCF-7 cell lines. Briefly, cells were incubated in 96-well plates at an approximate density of 5.0 × 10^3^ cells/well with 100 μL DMEM medium supplemented with 5% FBS, penicillin (100 U/mL) and streptomycin (100 μg/mL) at 37 °C with 5% CO_2_ for 24 h. Then, cells were fed with different concentrations of the test compounds (**1**–**12**) for another 48 h. After gently removing the culture media, 100 μL of MTT solution (0.5 mg/mL) was applied for staining the cells. Finally, absorbance of each plate was observed at 490 nm by the microplate reader after removal of MTT solution and addition of 100 μL dimethyl sulfoxide.

### 2.8. Tyrosinase Inhibitory Activity

l-Tyrosine was used as a substrate to evaluate the tyrosinase inhibition of **1**–**12** following the previously developed method [28,29] with some modifications. The tyrosinase inhibition assay was performed using 0.1 M phosphate buffer (pH 6.5), 2 mM l-tyrosine, and test compound solution with different concentrations ranging from 10 μM to 100 μM. The mixture was prepared 5 min before treatment with the enzyme solution (150 U/mL). In the experiments, the reaction mixture without tyrosinase was used as a blank, and the reaction mixture without sample solution was used as a negative control. Kojic acid was used as a positive control. After incubation for 30 min at 37 °C, absorbance of the reaction mixture was recorded at 490 nm to evaluate the amount of dopachrome produced in the process. The extent of tyrosinase inhibition by compounds **1**–**12** is displayed as the half maximal inhibitory concentration (IC_50_) in Table 2.

## 3. Results

Microbial transformation of three bioactive prenylchalcones **1**–**3** is reported herein. Fermentation of **1** with *R. oryzae* KCCM 60,556 furnished three new (**4**, **5**, and **7**) and one known (**6**) metabolites. Fermentation of **2** with *M. hiemalis* KCTC 26,779 furnished two known (**8** and **9**) metabolites. Fermentation of **3** with *M. hiemalis* KCTC 26,779 furnished one new (**10**) metabolite, while that with *M. ramanniana* var. *angulispora* KCTC 6137 resulted in the production of two known (**11** and **12**) metabolites (Figure 1) (Figures S8–S41).

Compound **4** was obtained as a yellow solid. High resolution electrospray ionization mass spectrometry (HRESIMS) measurements on **4** revealed a molecular formula of C_26_H_32_O_10_ corresponding to 11 unsaturation by the occurrence of its HRESIMS ion peak at *m*/*z* 527.1892 [M+Na]^+^ (calcd. for C_26_H_32_O_10_Na, 527.1893). The typical ultraviolet (UV) absorption at 371 nm indicated the appearance of a chalcone scaffold [4,30]. Inspection of its spectroscopic data revealed the presence of characteristic resonances for a chalcone skeleton with a 3-hydroxy-3-methylbutyl group and a sugar moiety. The ^1^H-NMR data presented signals assignable to two *ortho*-coupled aromatic protons at δ_H_ 7.98 (1H, d, *J* = 9.0) and 6.80 (1H, d, *J* = 9.0 Hz) and four aromatic protons at δ_H_ 7.64 (2H, d, *J* = 8.6 Hz) and 6.85 (2H, d, *J* = 8.6 Hz). Meanwhile, an olefin moiety was identified by the observation of signals at δ_H_ 7.82 (1H, d, *J* = 15.5) and 7.66 (1H, d, *J* = 15.5). These typical resonances suggested the existence of a 4,2′,3′,4′-tetrasubstituted chalcone. The two multiplets at δ_H_ 2.81 (2H) and 1.66 (2H) and two singlets at δ_H_ 1.28 (3H) and 1.26 (3H) were attributed to the 3-hydroxy-3-methylbutyl group, whereas the anomeric proton at δ_H_ 5.01 (1H, d, *J* = 7.5 Hz), together with the characteristic carbon signals at δ_C_ 102.0, 78.5, 78.2, 75.0, 71.4, and 62.6 demonstrated that the sugar moiety was a *β*-d-glucopyranose [31,32]. Significant heteronuclear multiple bond coherence (HMBC) correlation between the anomeric proton signal at δ_H_ 5.00 (H-1′′′) and δ_C_ 162.6 (C-4′) indicated that the *β*-d-glucose was attached to the C-4′ position, while the 3-hydroxy-3-methylbutyl group was located at C-3′ on the basis of the HMBC correlations between δ_H_ 2.81 (H-1′′)/1.66 (H-2′′) and δ_c_ 120.8 (C-3′). Furthermore, acid hydrolysis of **4** produced d-glucose which was confirmed through direct comparison with the standard on TLC. Thus, the structure of **4** was characterized as 3′-(3-hydroxy-3-methylbutyl)-4′-*O*-β-d-glucopyranosyl-4,2′-dihydroxychalcone.

Compound **5** was assigned the composition C_27_H_34_O_10_ from its HRESIMS at *m*/*z* 541.2049 [M+Na]^+^ (calcd. for C_27_H_34_O_10_Na, 541.2050). The ^1^H- and ^13^C-NMR spectra of **5** were quite similar to those of **4**, except for the resonances of a methoxy group (δ_H_ 3.31/δ_C_ 49.8) in **5**. The attachment of the methoxy group was determined to be at C-3′′ according to the HMBC correlations from the methoxy protons H-6′′ (δ_H_ 3.31) to C-3′′ (δ_C_ 76.9) and C-2′′ (δ_C_ 39.2). Thus, the structure of **5** was determined as 3′-(3-*O*-methyl-3-methylbutyl)-4′-*O*-β-d-glucopyranosyl-4,2′-dihydroxychalcone.

**Figure 2:**
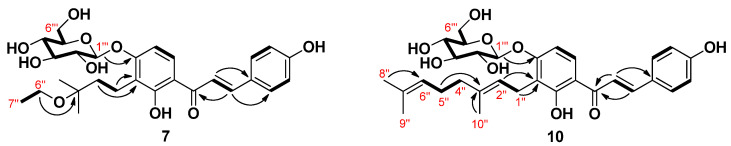
Key COSY (^1^H-^1^H) and HMBC (^1^H→^13^C) correlations of compounds **7** and **10**.

The molecular formula of compound **7** was determined as C_28_H_36_O_10_ using HRESIMS on the basis of a molecular ion at *m*/*z* 555.2208 [M+Na]^+^ (calcd. for C_28_H_36_O_10_Na,555.2206), which corresponded to 11 degrees of unsaturation. The NMR data of **7** were almost identical to those observed for **4** except for the appearance of one *O*-methylene (δ_H_ 3.55, 2H, q, *J* = 7.0 Hz/δ_C_ 58.0) and one methyl (δ_H_ 1.21, 3H, t, *J* = 7.0 Hz/δ_C_ 16.5) signals corresponding to H-6′’ and H-7′’, readily attributable to an ethyloxy group. Further evidence was obtained from the obvious correlation between proton signals H-6′′ and H-7′′ (Figure 2) in the ^1^H-^1^H homonuclear correlation spectroscopy (COSY) experiment. HMBC correlation between H-6′’ and C-3′’ confirmed the location of the ethyloxy group at C-3′’. Accordingly, the structure of **7** was assigned 3′-(3-*O*-ethyl-3-methylbutyl)-4′-*O*-β-d-glucopyranosyl-4,2′-dihydroxychalcone.

Compound **10** was confirmed to have C_31_H_38_O_9_ as the elemental composition according to the analysis of its HRESIMS data. The ^1^H-NMR spectrum of compound **10** displayed two sets of aromatic proton resonances at δ_H_ 8.21 (1H, d, *J* = 9.2) and 6.74 (1H, d, *J* = 9.2), 7.79 (2H, d, *J* = 8.5), and 6.85 (2H, d, *J* = 8.5) together with two olefinic proton resonances at δ_H_ 7.84 (1H, d, *J* = 15.3) and 7.80 (1H, d, *J* = 15.3) assignable to the 4,2′,3′,4′-tetrasubstituted chalcone. In addition, proton resonance signals for a geranyl group and a sugar moiety were identified in the up-field region. Analysis of the COSY spectrum together with the heteronuclear single quantum coherence (HSQC) and HMBC spectra allowed the establishment of the structure as 4,2′,4′-trihydroxychalcone substituted with a geranyl group and a sugar moiety in **10** (Figure 2). The ^13^C-NMR data showed six signals at δ_C_ 100.1, 77.3, 76.8, 73.4, 69.8, and 60.7, which are characteristic of a glucose moiety [31]. The anomeric proton signal at δ_H_ 5.00 (1H, d, *J* = 7.3 Hz) indicated a β-configuration of the glucosidic bond. The connectivity of the glucose moiety in **10** was confirmed to be at C-4′ through an ether linkage by the HMBC correlation from the anomeric proton signal H-1′’’ to C-4′. Presence of the glucose moiety and its aglycone after acid hydrolysis was further confirmed by comparison of their TLC and HPLC identification pattern with those of the authentic samples. Linkage of the geranyl group was deduced to be at C-3′ position by the observation of HMBC correlations from H-1′′/H-2′′ to C-3′. Hence, **10** was assigned 4′-*O*-β-d-glucopyranosyl xanthoangelol.

Structures of the five known metabolites were identified as 3′-(3-methyl-2-butenyl)-4′-*O*-β-d-glucopyranosyl-4,2′-dihydroxychalcone (**6**) [33], xanthoangelols H (**8**) [34,35] and D (**9**) [36], xanthokeismin A (**11**) [5], and xanthoangelol B (**12**) [25] by comparing ^1^H- and ^13^C-NMR data with those reported in the literature.

In this study, cytotoxicity of compounds **1**–**12** was determined by MTT assays against A375P, HT-29, and MCF-7 human cancer cells. As shown in Table 2, compounds **1** and **3** showed most potent cytotoxicity against A375P, HT-29, and MCF-7. Compound **2** exhibited moderate cytotoxic activity toward human cancer cell lines tested. However, all the metabolites **4**–**12** exhibited lower cytotoxicity compared with their corresponding substrates **1**, **2**, and **3**. It appears that the *C*-prenyl or *C*-geranyl side chain is essential for cytotoxic activity. Meanwhile, glucosylation or methylation at 4′-OH decreased the cytotoxicity.

The capability of **1**–**12** to inhibit tyrosinase was determined using l-tyrosine as a substrate. The most potent compounds were **5** and **7**, which displayed respectively 2.5- and 2-fold stronger inhibition compared with the positive control. Compound **4** showed moderate activity comparable to kojic acid. It was suggested that methyl or ethyl moiety introduced at the 3-*O* position of 3-hydroxy-3-methylbutyl group could improve the inhibitory activity against mushroom tyrosinase. The anti-tyrosinase potencies were enhanced in chalcone 4′-*O*-glucosides (**6** and **10**) compared with their corresponding aglycones (**1** and **3**). This allowed us to postulate that the introduction of 4′-*O*-glucopyranosyl group led to the improvement in the tyrosinase inhibitory capacity.

## 4. Discussion

In this work, three bioactive prenylchalcones, isobavachalcone (IBC, **1**), 4-hydroxyderricin (HD, **2**), and xanthoangelol (XT, **3**), were isolated from *A. keiskei* and subjected to microbial transformation. The reaction types consisted of hydroxylation, methylation, ethylation, rearrangement, and glucosylation. These highly specific reactions are difficult to achieve by synthetic methods, especially under mild conditions. Therefore, microbial transformation is a useful way in structural diversification of chalcones to discover valuable derivatives. In addition, microbial transformation permits the possibility of understanding the metabolic pathway [37]. It is particularly noteworthy that microbial transformation of HD (**2**) with the microbe *M. hiemalis* produced two metabolites xanthoangelols H (**8**) and D (**9**), while microbial transformation of XT (**3**) with the microbe *Mortierella ramanniana* var. *angulispora* resulted in the production of two metabolites xanthokeismin A (**11**) and xanthoangelol B (**12**). All the four known metabolites (**8**, **9**, **11**, and **12**) were previously identified from the herb *A. keiskei*. The information could be beneficial for understanding the metabolic pathways of these compounds in this plant.

**Figure 3:**
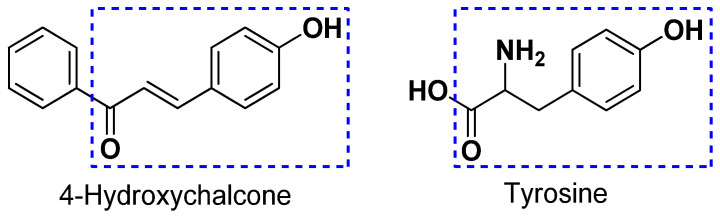
The structural similarity between 4-hydroxychalcone and tyrosine.

Tyrosinase is a metalloenzyme containing two copper atoms and acts as a rate-limiting oxidase involved in the production of melanin [38]. During melanogenesis, tyrosinase is able to act as a hydroxylase to catalyze the conversion of l-tyrosine to l-3,4-dihydroxyphenylalanine (l-DOPA) and subsequently is able to act as an oxidase to catalyze the conversion of l-DOPA to l-dopaquinone, which results in the accumulation of melanin and hyperpigmentation [39]. In recent years, it has been demonstrated that various dermatological disorders, such as age spots, melasma freckle, and sites of actinic damage, derive from the accumulation of an exaggerated level of epidermal pigmentation. Tyrosinase has been recognized as a significant target for the treatment of skin disorders related to irregular pigmentation [40]. Thus, the identification of new and potent leads with tyrosinase inhibitory activity has attracted considerable interest in medication and cosmetics. Chalcones represent a diverse class of compounds generally presented in higher plants. Structurally, compounds with the scaffold of 4-hydroxychalcone are considered as potent tyrosinase inhibitors owing to the structural similarity between 4-hydroxychalcone and tyrosine (Figure 3) [41]. In this study, the structure -activity relationship of twelve compounds (**1**–**12**) with the scaffold of 4-hydroxychalcone was investigated for its tyrosinase inhibitory activity. The results revealed that the glucosylation of the OH group at C-4′ position is important in the enhancement of the inhibition. Furthermore, the insertion of a methyl (compound **5**) or an ethyl group (compound **7**) into the 3-hydroxy-3-methylbutyl group at the 3-*O* position dramatically improved the inhibition. Considering that **5** and **7** showed the most potent tyrosinase inhibition with relatively low cytotoxicity, as shown in Table 2, they can act as potential leads for the development of effective and safe anti-browning and skin-whitening agents.

## 5. Conclusions

Microbial transformation of three bioactive prenylchalcones **1**–**3** isolated from *A. keiskei* afforded four new (**4**, **5**, **7**, and **10**) and five known (**6**, **8**, **9**, **11**, and **12**) metabolites. The five known metabolites were all previously reported from natural sources. In the evaluation of all compounds for their cytotoxic and tyrosinase inhibitory activities, metabolites **5** and **7** exhibited the most potent tyrosinase inhibition with relatively low cytotoxicity. The two metabolites **5** and **7** can be considered as new leads for the further design of tyrosinase inhibitors. The present results indicate that microbial transformation can be an efficient and useful tool for the identification of natural product-like compounds as bioactive leads.

## Figures and Tables

**Figure 1:**
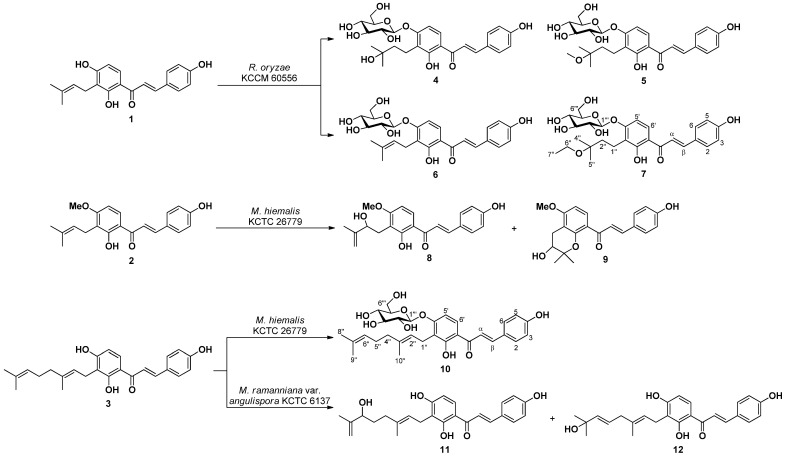
Compounds **4**–**12** obtained by microbial transformation of **1**–**3**.

**Table 1:** ^1^H- and ^13^C-NMR data for **4**, **5**, **7**, and **10**.

	4 ^a^		5 ^a^		7 ^a^		10 ^b^	
C/H	δ_H_ (*J*/Hz)	δ_C_	δ_H_ (*J*/Hz)	δ_C_	δ_H_ (*J*/Hz)	δ_C_	δ_H_ (*J*/Hz)	δ_C_
α	7.66 d (15.5)	118.4	7.67 d (15.2)	118.4	7.65 d (15.3)	118.4	7.80 d (15.3)	117.1
β	7.82 d (15.5)	146.4	7.84 d (15.2)	146.4	7.83 d (15.3)	146.4	7.84 d (15.3)	145.0
C = O		194.5		194.5		194.5		192.5
1		127.9		127.9		127.9		125.6
2,6	7.64 d (8.6)	132.1	7.66 d (8.6)	132.1	7.64 d (8.6)	132.1	7.79 d (8.5)	131.5
3,5	6.85 d (8.6)	117.1	6.87 d (8.6)	117.1	6.85 d (8.6)	117.1	6.85 d (8.5)	115.9
4		161.9		161.9		161.9		160.6
1′		116.6		116.6		116.6		114.5
2′		164.1		164.2		164.2		162.4
3′		120.8		120.5		120.7		117.1
4′		162.6		162.6		162.6		161.0
5′	6.80 d (9.0)	107.2	6.82 d (9.1)	107.1	6.80 d (9.0)	107.1	6.74 d (9.2)	105.9
6′	7.98 d (9.0)	130.6	8.00 d (9.1)	130.6	7.98 d (9.0)	130.6	8.21 d (9.2)	130.6
1′′	2.81 m	18.6	2.78 m	18.2	2.77 m	18.3	3.44 m, 3.24 m	21.4
2′’	1.66 m	43.2	1.71 m	39.2	1.69 m	39.7	5.22 m	121.9
3′′		72.0		76.9		76.7		134.3
4′′	1.28 s	28.7	1.27 s	25.1	1.26 s	25.7	1.90 m	39.3
5′′	1.26 s	29.8	1.25 s	25.8	1.24 s	26.6	1.99 m	26.2
6′′			3.31 s	49.8	3.55 q (7.0)	58.0	5.04 t (7.0)	124.2
7′′					1.21 t (7.0)	16.5		129.9
8′′							1.59 s	25.5
9′′							1.53 s	17.5
10′′							1.75 s	16.0
1′’’	5.01 d (7.5)	102.0	5.06 d (7.3)	101.9	5.04 d (7.3)	102.0	5.00 d (7.3)	100.1
2′′′	3.53 m	75.0	3.52 m	75.1	3.51 m	75.1	3.32 m	73.4
3′′′	3.49 m	78.5	3.51 m	78.5	3.49 m	78.5	3.39 m	77.3
4′′′	3.41 m	71.4	3.42 m	78.4	3.41 m	71.4	3.18 m	69.8
5′′′	3.49 m	78.2	3.50 m	71.4	3.49 m	78.3	3.32 m	76.8
6′′′	3.92 dd (12.1, 2.0) 3.72 dd (12.1, 5.0)	62.6	3.93 dd (12.1, 2.1) 3.74 dd (12.1, 5.9)	62.7	3.92 dd (12.3, 2.1) 3.72 dd (12.3, 5.3)	62.7	3.71 m 3.46 m	60.7

Assignments were achieved on the basis of 1D and 2D NMR data. The values of *J* are presented in parentheses. ^a^ Data were acquired in methanol-*d*_4_. ^b^ Data were acquired in DMSO-*d*_6_.

**Table 2:** Cytotoxic and tyrosinase inhibitory activities of **1**–**12** ^a^.

Compound	IC_50_ ± SD (μM)			
	A375P	HT-29	MCF-7	Tyrosinase
**1**	9.78 ± 1.06	26.33 ± 2.42	24.02 ± 0.36	>100
**2**	25.34 ± 0.66	37.30 ± 4.86	29.71 ± 2.78	>100
**3**	13.00 ± 1.28	24.35 ± 1.27	21.92 ± 2.30	>100
**4**	>80	>80	>80	78.47 ± 3.13
**5**	77.96 ± 3.44	>80	>80	28.68 ± 1.24
**6**	>80	>80	>80	43.12 ± 6.74
**7**	>80	>80	>80	33.32 ± 1.16
**8**	29.65 ± 2.09	54.36 ± 1.03	32.44 ± 0.55	>100
**9**	21.18 ± 0.20	58.55 ± 4.19	28.18 ± 1.34	52.65 ± 2.85
**10**	29.03 ± 2.77	>80	>80	71.71 ± 9.22
**11**	69.77 ± 3.88	>80	>80	>100
**12**	20.56 ± 2.17	>80	59.01 ± 2.41	>100
**DZ**	2.52 ± 0.23	8.68 ± 0.90	2.87 ± 0.38	
**Kojic acid**				77.74 ± 2.55

^a^ Results are expressed as the mean values of three experiments ± SD.

## Data Availability

Not applicable.

## Supplementary Materials

The following supporting information can be downloaded at: https://www.mdpi.com/article/10.3390/foods11040543/s1, Figure S1: TLC analyses for microbial transformation of **1**–**3** by selected microbes, Figures S2–S41: 1D, 2D NMR, and HRESIMS spectra of **1**–**12**.
