# Supplementary material for: Effects of Microbial Transformation on the Biological Activities of Prenylated Chalcones from Angelica keiskei
Source: Foods. 2022 Feb 14;11(4):543. doi: 10.3390/foods11040543 (PMC8871312; doi:10.3390/foods11040543)
Supplement: Supplementary file 1 [file foods-11-00543-s001.zip › foods-1593754-supplementary.pdf]

Supplementary Material

Effects of Microbial Transformation on the Biological Activities of Prenylated Chalcones from *Angelica keiskei*

Yina Xiao, Ik-Soo Lee *

College of Pharmacy, Chonnam National University, Gwangju 61186, Korea; yogurtxiao@163.com

* Correspondence: islee@chonnam.ac.kr; Tel.: +82-62-530-2932

Contents of Supplementary Material

Figure S1. TLC analyses for microbial transformation of **1-3** by selected microbes 4

Figure S2. ^{1}H-NMR (400 MHz, methanol-d_4) spectrum of **1** 5

Figure S3. ^{13}C-NMR (100 MHz, methanol-d_4) spectrum of **1** 5

Figure S4. ^{1}H-NMR (400 MHz, acetone-d_6) spectrum of **2** 6

Figure S5. ^{13}C-NMR (100 MHz, acetone-d_6) spectrum of **2** 6

Figure S6. ^{1}H-NMR (400 MHz, acetone-d_6) spectrum of **3** 7

Figure S7. ^{13}C-NMR (100 MHz, acetone-d_6) spectrum of **3** 7

Figure S8. ^{1}H-NMR (500 MHz, methanol-d_4) spectrum of **4** 8

Figure S9. ^{13}C-NMR (125 MHz, methanol-d_4) spectrum of **4** 8

Figure S10. HSQC spectrum of **4** 9

Figure S11. HMBC spectrum of **4** 9

Figure S12. COSY spectrum of **4** 10

Figure S13. HRESIMS spectrum of **4** 11

Figure S14. ^{1}H-NMR (600 MHz, methanol-d_4) spectrum of **5** 12

Figure S15. ^{13}C-NMR (150 MHz, methanol-d_4) spectrum of **5** 12

Figure S16. HSQC spectrum of **5** 13

Figure S17. HMBC spectrum of **5** 13

Figure S18. COSY spectrum of **5** 14

Figure S19. HRESIMS spectrum of **5** 15

Figure S20. ^{1}H-NMR (500 MHz, methanol-d_4) spectrum of **7** 16

Figure S21. ^{13}C-NMR (125 MHz, methanol-d_4) spectrum of **7** 16

Figure S22. HSQC spectrum of **7** 17

Figure S23. HMBC spectrum of **7** 17

Figure S24. COSY spectrum of **7** 18

Figure S25. HRESIMS spectrum of **7** 19

Figure S26. ^{1}H-NMR (600 MHz, DMSO-d_6) spectrum of **10** 20

Figure S27. ^{13}C-NMR (150 MHz, DMSO-d_6) spectrum of **10** 20

Figure S28. HSQC spectrum of **10** 21

Figure S29. HMBC spectrum of **10** 21

Figure S30. COSY spectrum of **10** ... 22

Figure S31. HRESIMS spectrum of **10** ... 23

Figure S32. ^{1}H-NMR (500 MHz, methanol-d_4) spectrum of **6** ... 24

Figure S33. ^{13}C-NMR (125 MHz, methanol-d_4) spectrum of **6** ... 24

Figure S34. ^{1}H-NMR (500 MHz, methanol-d_4) spectrum of **8** ... 25

Figure S35. ^{13}C-NMR (100 MHz, methanol-d_4) spectrum of **8** ... 25

Figure S36. ^{1}H-NMR (500 MHz, methanol-d_4) spectrum of **9** ... 26

Figure S37. ^{13}C-NMR (125 MHz, methanol-d_4) spectrum of **9** ... 26

Figure S38. ^{1}H-NMR (500 MHz, acetone-d_6) spectrum of **11** ... 27

Figure S39. ^{13}C-NMR (125 MHz, acetone-d_6) spectrum of **11** ... 27

Figure S40. ^{1}H-NMR (600 MHz, methanol-d_4) spectrum of **12** ... 28

Figure S41. ^{13}C-NMR (150 MHz, methanol-d_4) spectrum of **12** ... 28

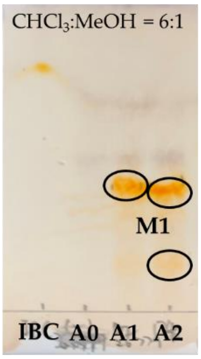

IBC (Isobavachalcone): Substrate control
A0: Culture control at 4 days
A1: IBC-added culture *Mucor plumbeus* extract at 2 days
A2: IBC-added culture *Mucor plumbeus* extract at 4 days
M1: Potential metabolites

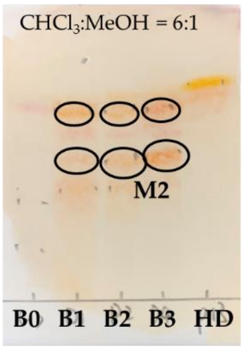

HD (4-Hydroxyderricin): Substrate control
B0: Culture control at 6 days
B1: HD-added culture *Mucor hiemalis* extract at 2 days
B2: HD-added culture *Mucor hiemalis* extract at 4 days
B3: HD-added culture *Mucor hiemalis* extract at 6 days
M2: Potential metabolites

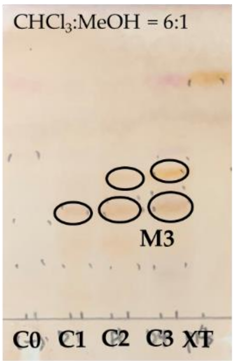

XT (xanthoangelol): Substrate control
B0: Culture control at 6 days
B1: XT-added culture *Mucor hiemalis* extract at 2 days
B2: XT-added culture *Mucor hiemalis* extract at 4 days
B3: XT-added culture *Mucor hiemalis* extract at 6 days
M3: Potential metabolites

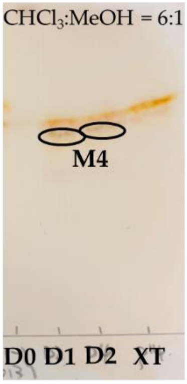

XT (xanthoangelol): Substrate control
D0: Culture control at 4 days
D1: XT-added culture *Mortierella ramanniana var. angulispora* extract at 2 days
D2: XT-added culture *Mortierella ramanniana var. angulispora* extract at 4 days
M4: Potential metabolites

Figure S1. TLC analyses for microbial transformation of **1**-**3** by selected microbes

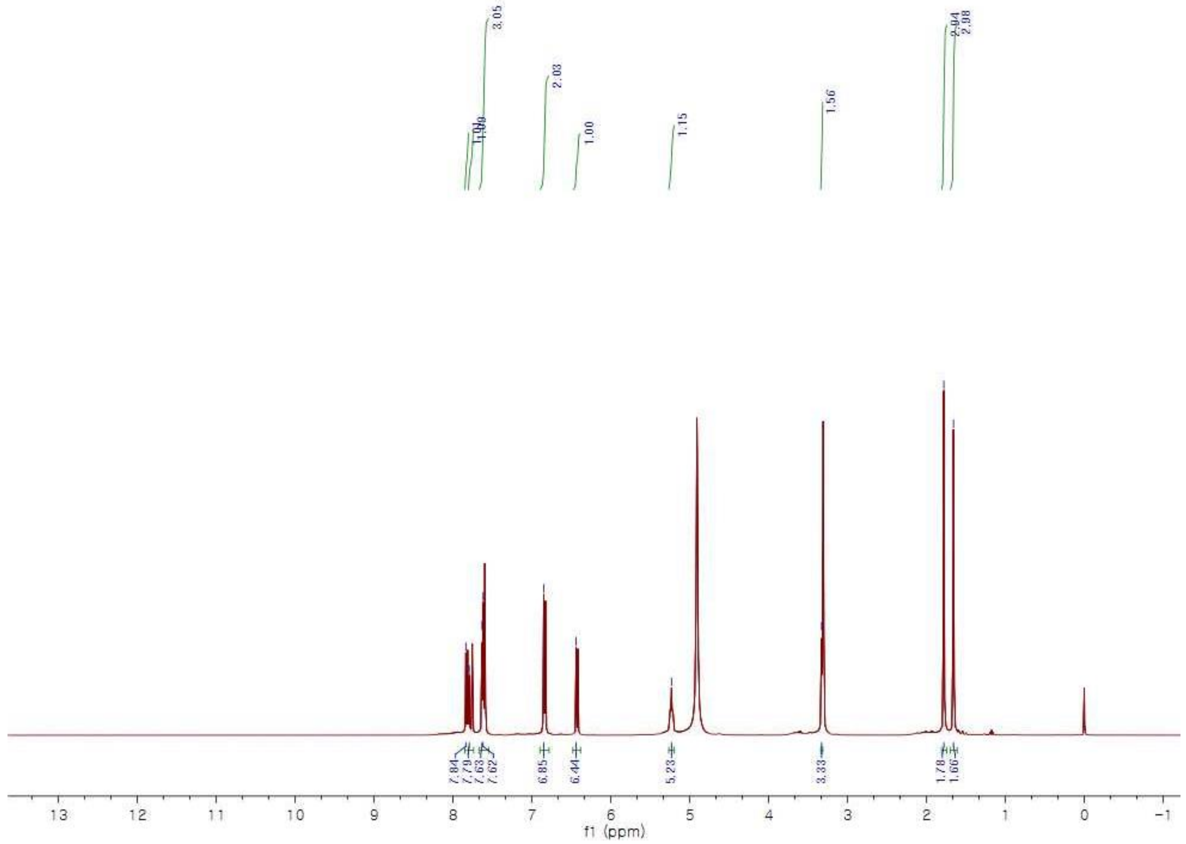

Figure S2. ^{1}H-NMR (400 MHz, methanol-d_4) spectrum of **1**

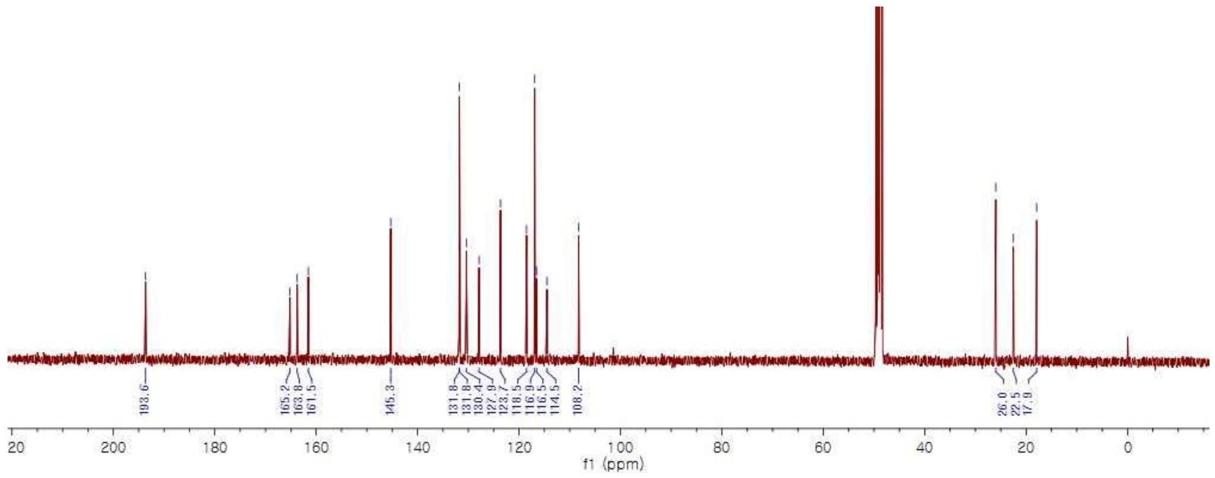

Figure S3. ^{13}C-NMR (100 MHz, methanol-d_4) spectrum of **1**

Isobavachalcone (**1**): ^{1}H-NMR (methanol-d_4, 400 MHz, δ in ppm, *J* in Hz) δ 7.84 (1H, d, *J* = 8.9, H-6′), 7.79 (1H, d, *J* = 15.4, H-β), 7.63 (1H, d, *J* = 15.4, H-α), 7.62 (2H, d, *J* = 8.8, H-2,6), 6.85 (2H, d, *J* = 8.8, H-3,5), 6.44 (1H, d, *J* = 8.9, H-5′), 5.23 (1H, m, H-2′′), 3.33 (2H, d, *J* = 7.2, H-1′′), 1.78 (3H, s, H-4′′), 1.66 (3H, s, H-5′′); ^{13}C-NMR (methanol-d_4, 100 MHz, δ in ppm): 193.6 (C=O), 165.2 (C-4′), 163.8 (C-2′), 161.5 (C-4), 145.3 (C-β), 131.8 (C-2,6), 131.8 (C-3′′), 130.4 (C-6′), 127.9 (C-1), 123.7 (C-2′′), 118.5 (C-α), 116.9 (C-3,5), 116.5 (C-3′), 114.5 (C-1′), 108.2 (C-5′), 26.0 (C-4′′), 22.5 (C-1′′), 17.9 (C-5′′).

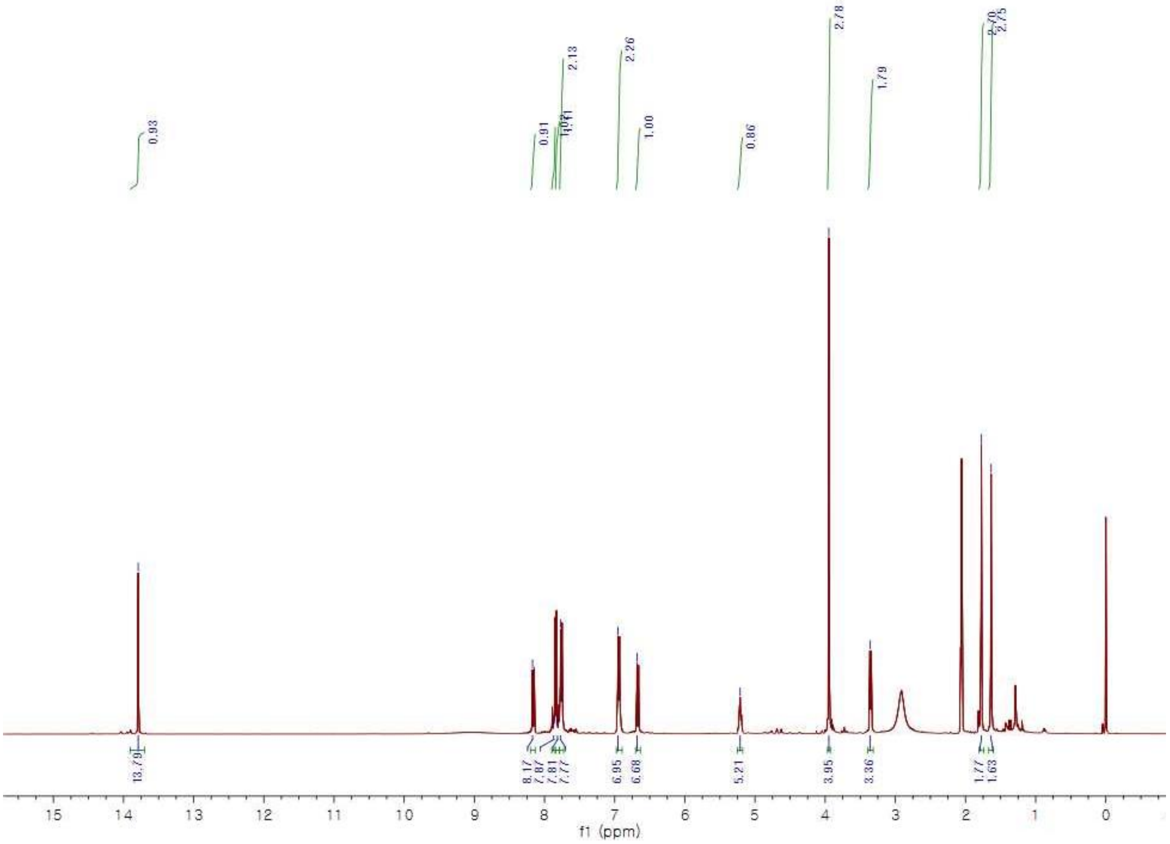

Figure S4. ^{1}H-NMR (400 MHz, acetone-d_6) spectrum of **2**

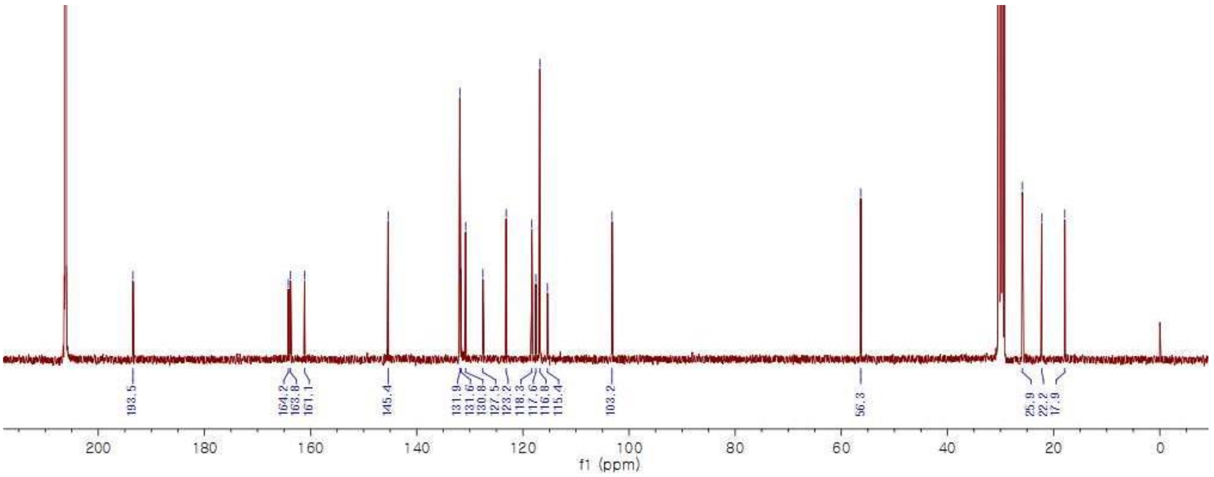

Figure S5. ^{13}C-NMR (100 MHz, acetone-d_6) spectrum of **2**

4-Hydroxyderricin (**2**): ^{1}H-NMR (acetone-d_6, 400 MHz, δ in ppm, J in Hz) δ 13.79 (1H, s, OH), 8.17 (1H, d, J = 9.0, H-6′), 7.87 (1H, d, J = 15.4, H-β), 7.81 (1H, d, J = 15.4, H-α), 7.77 (2H, d, J = 8.5, H-2,6), 6.95 (2H, d, J = 8.5, H-3,5), 6.68 (1H, d, J = 9.0, H-5′), 5.21 (1H, m, H-2′′), 3.95 (3H, s, OMe), 3.36 (2H, d, J = 7.2, H-1′′), 1.77 (3H, s, H-4′′), 1.63 (3H, s, H-5′′); ^{13}C-NMR (acetond-d_6, 100 MHz, δ in ppm): 193.5 (C=O), 164.2 (C-4′), 163.8 (C-2′), 161.1 (C-4), 145.4 (C-β), 131.9 (C-2,6), 131.6 (C-3′′), 130.8 (C-6′), 127.5 (C-1), 123.2 (C-2′′), 118.3 (C-α), 117.6 (C-3′), 116.8 (C-3,5), 115.4 (C-1′), 103.2 (C-5′), 56.8 (OMe), 25.9 (C-4′′), 22.1 (C-1′′), 17.9 (C-5′′).

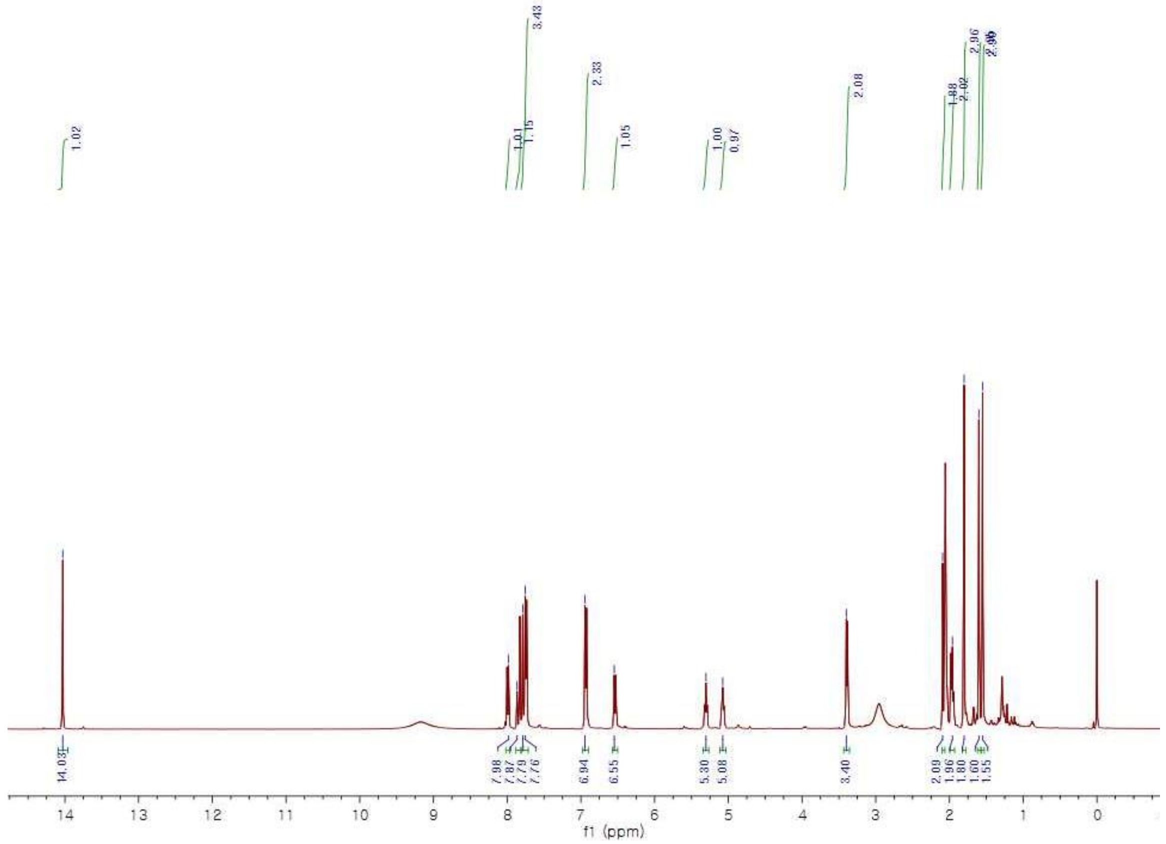

Figure S6. ^{1}H-NMR (400 MHz, acetone-d_6) spectrum of **3**

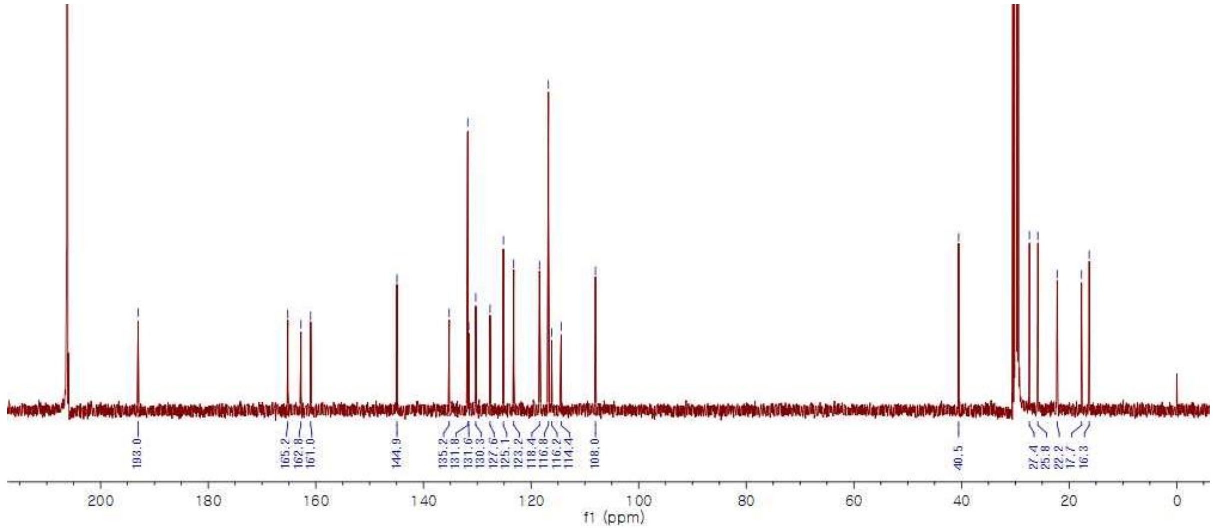

Figure S7. ^{13}C-NMR (100 MHz, acetone-d_6) spectrum of **3**

Xanthoangelol (**3**): ^{1}H-NMR (acetone-d_6, 400 MHz, δ in ppm, *J* in Hz) δ 14.03 (1H, s, OH), 7.98 (1H, d, *J* = 8.8, H-6′), 7.87 (1H, d, *J* = 15.2, H-β), 7.79 (1H, d, *J* = 15.2, H-α), 7.76 (2H, d, *J* = 8.5, H-2,6), 6.94 (2H, d, *J* = 8.5, H-3,5), 6.55 (1H, d, *J* = 8.8, H-5′), 5.30 (1H, m, H-2′′), 5.08 (1H, m, H-6′′), 3.40 (2H, d, *J* = 7.1, H-1′′), 2.09 (2H, overlapped, H-5′′), 1.96 (2H, m, H-4′′), 1.80 (3H, s, H-10′′), 1.60 (3H, s, H-8′′), 1.55 (3H, s, H-9′′); ^{13}C-NMR (acetone-d_6, 100 MHz, δ in ppm): 193.0 (C=O), 165.2 (C-4′), 162.8 (C-2′), 161.0 (C-4), 144.9 (C-β), 135.2 (C-3′′), 131.8 (C-2,6), 131.6 (C-7′′), 130.3 (C-6′), 127.6 (C-1), 125.1 (C-6′′), 123.2 (C-2′′), 118.4 (C-α), 116.8 (C-3,5), 116.2 (C-3′), 114.4 (C-1′), 108.0 (C-5′), 40.5 (C-4′′), 27.4 (C-5′′), 25.8 (C-8′′), 22.2 (C-1′′), 17.7 (C-9′′), 16.3 (C-10′′).

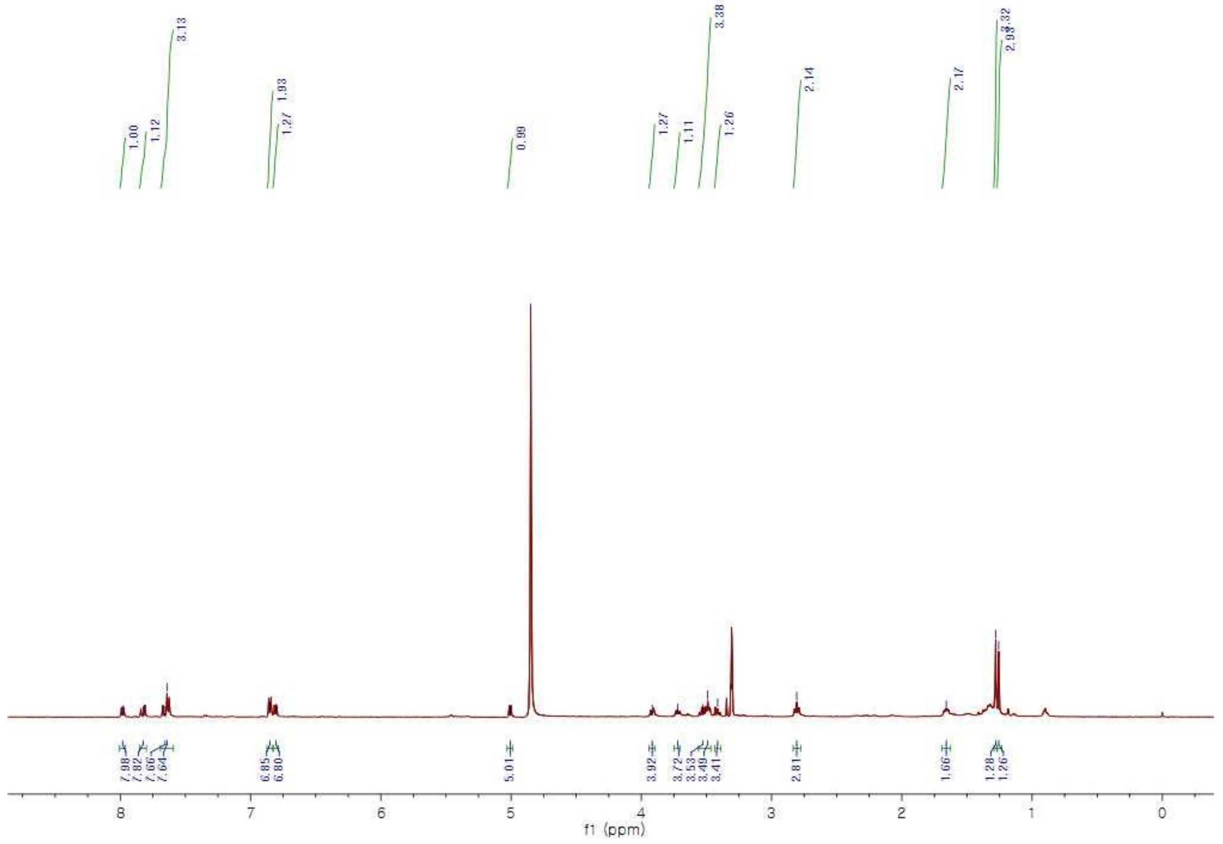

Figure S8. ^{1}H-NMR (500 MHz, methanol-d_4) spectrum of **4**

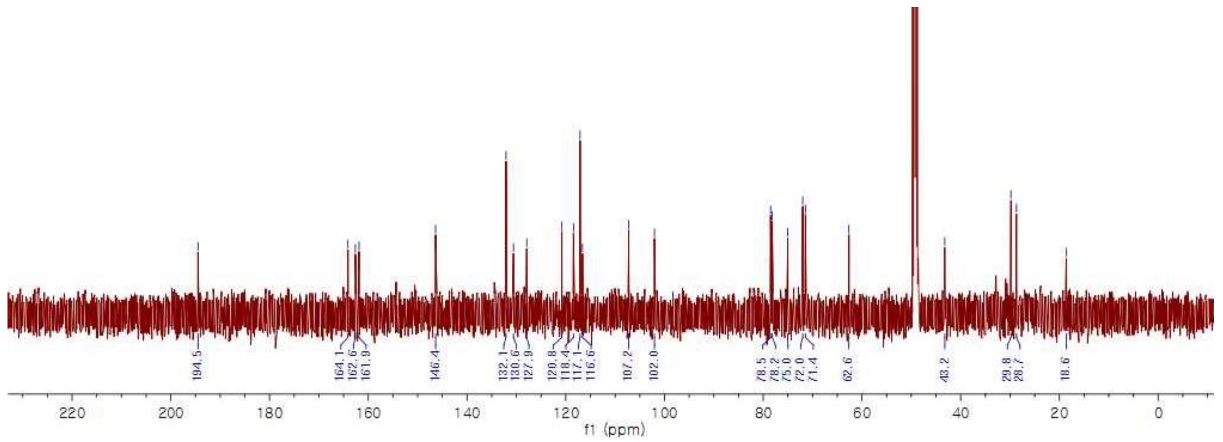

Figure S9. ^{13}C-NMR (125 MHz, methanol-d_4) spectrum of **4**

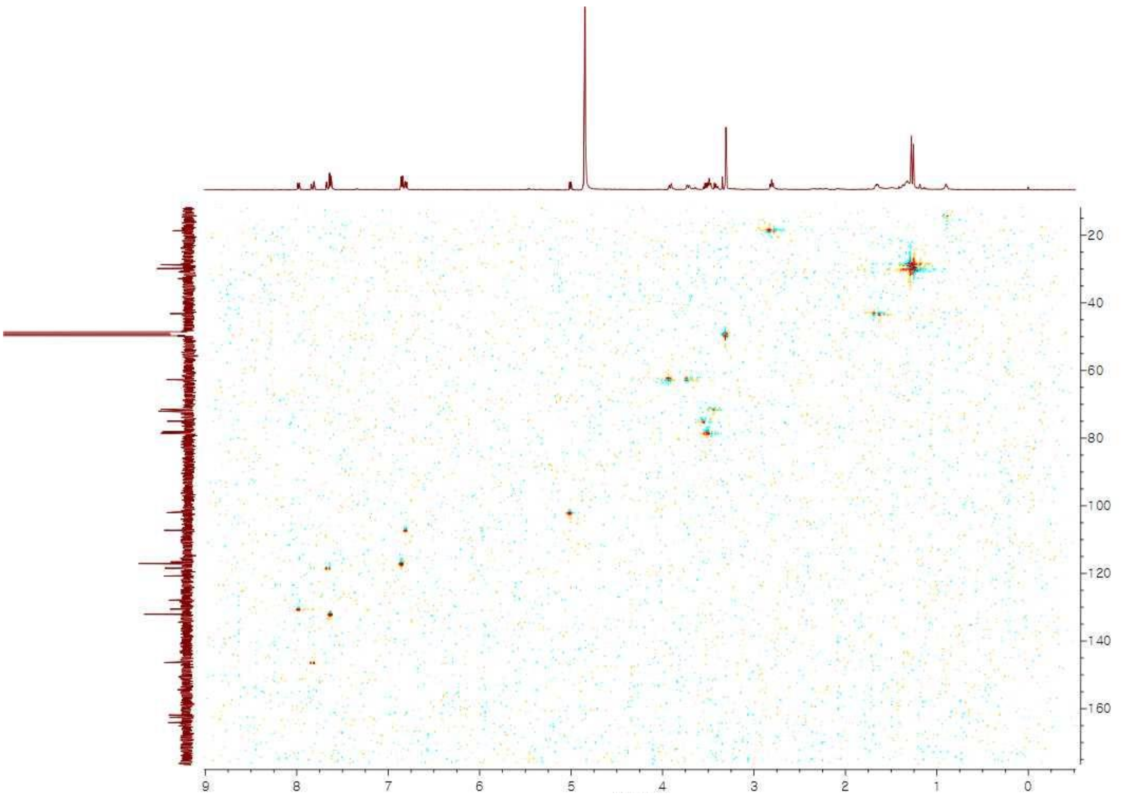

Figure S10. HSQC spectrum of **4**

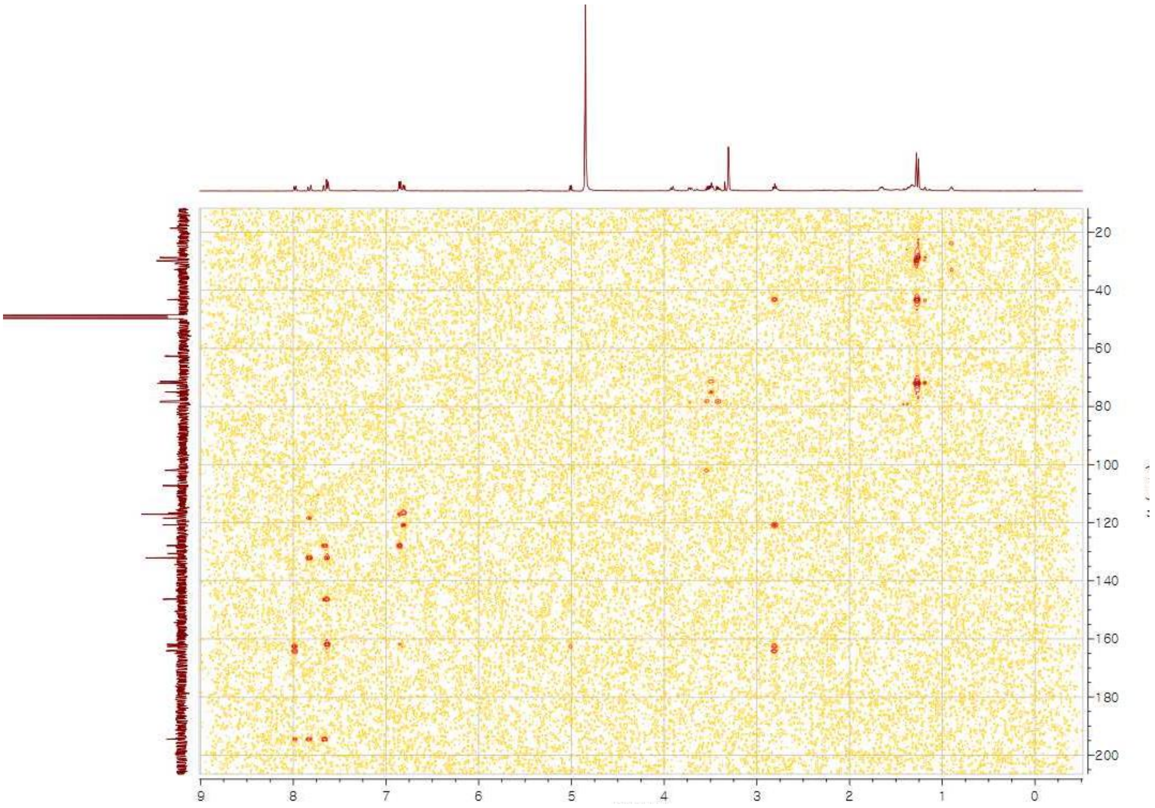

Figure S11. HMBC spectrum of **4**

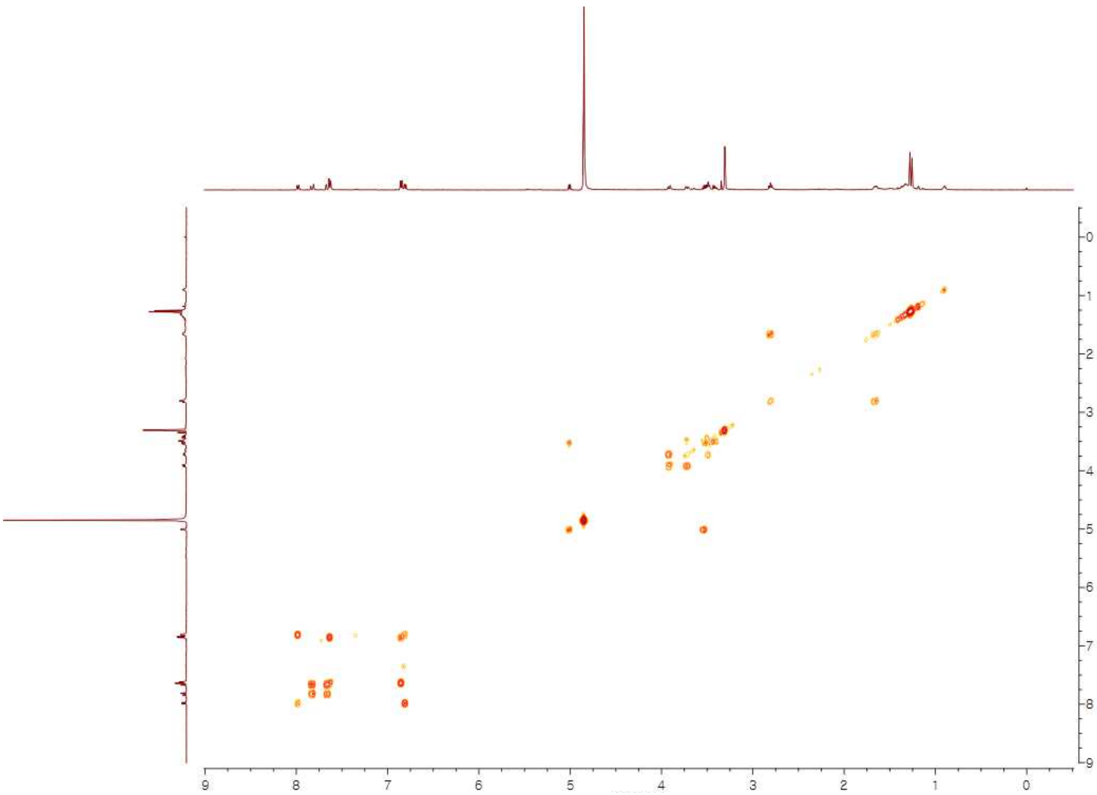

Figure S12. COSY spectrum of **4**

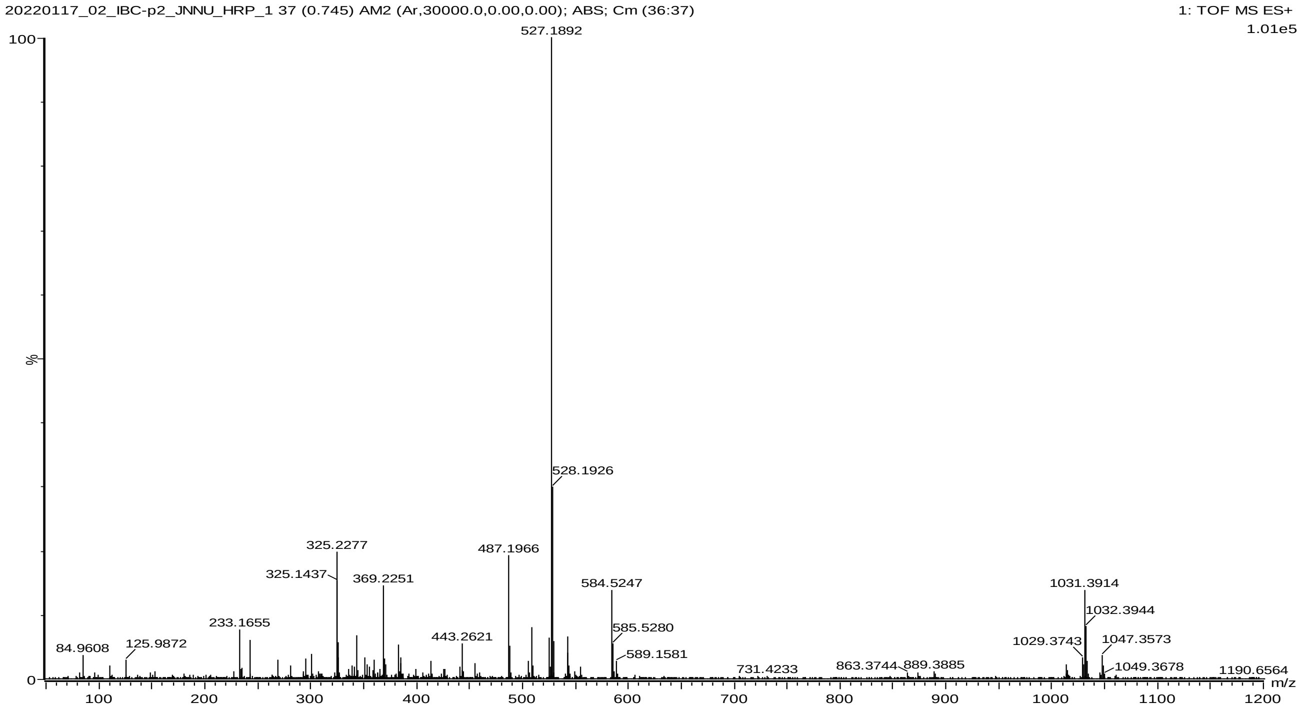

Elemental Composition Report

Single Mass Analysis
Tolerance = 5.0 PPM / DBE: min = -3.0, max = 200.0
Element prediction: Off
Number of isotope peaks used for i-FIT = 3

Monoisotopic Mass, Even Electron Ions
Elements Used:
C: 0-35 H: 0 -50 O: 0 -10 Na: 0 -1

Minimum:				-3.0				
Maximum:		300.0	5.0	200.0				
Mass	Calc. Mass	mDa	PPM	DBE	i-FIT	Norm	Conf(%)	Formula
525.1732	525.1737	-0.5	-1.0	11.5	476.2	n/a	n/a	C26 H30 O10 Na
527.1892	527.1893	-0.1	-0.2	10.5	572.9	0.005	99.47	C26 H32 O10 Na
	527.1917	-2.5	-4.7	13.5	578.2	5.235	0.53	C28 H31 O10

Figure S13. HRESIMS spectrum of **4**

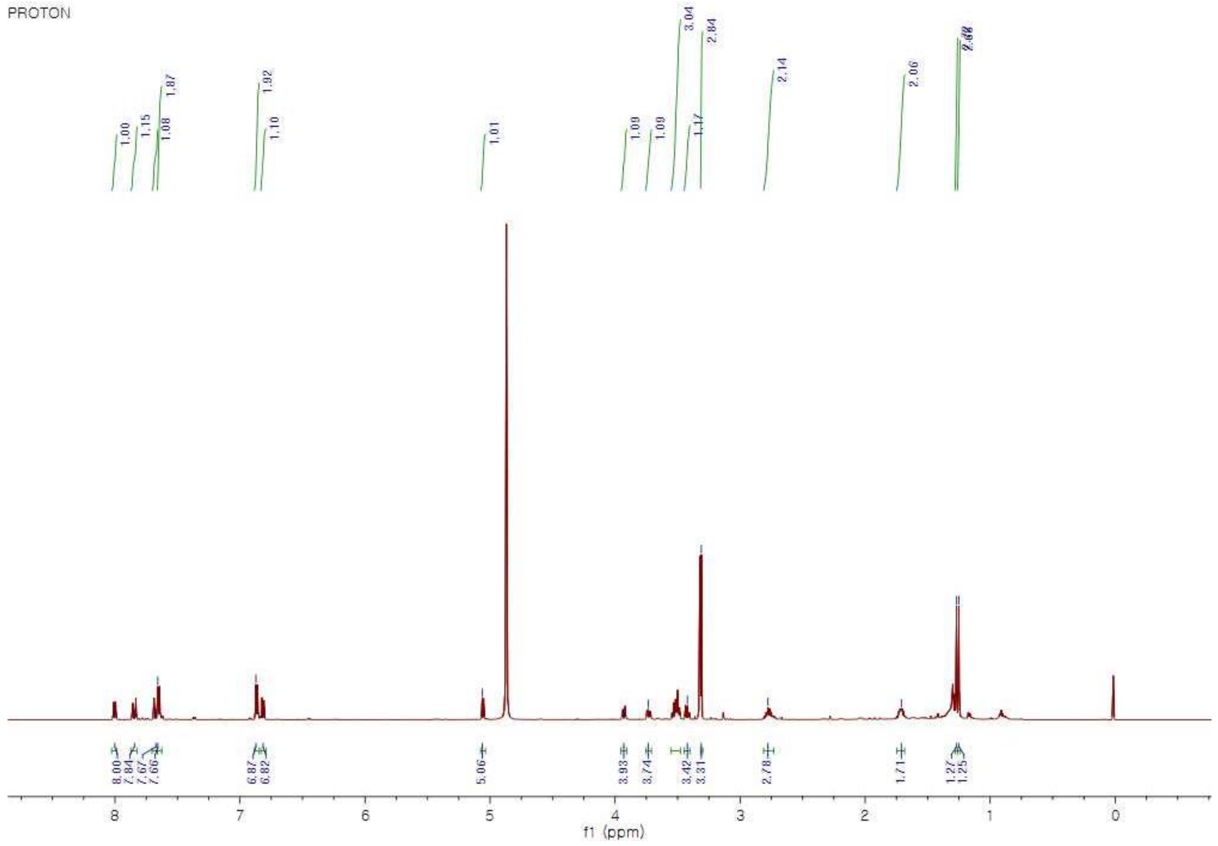

Figure S14. ¹H-NMR (600 MHz, methanol-d_4) spectrum of **5**

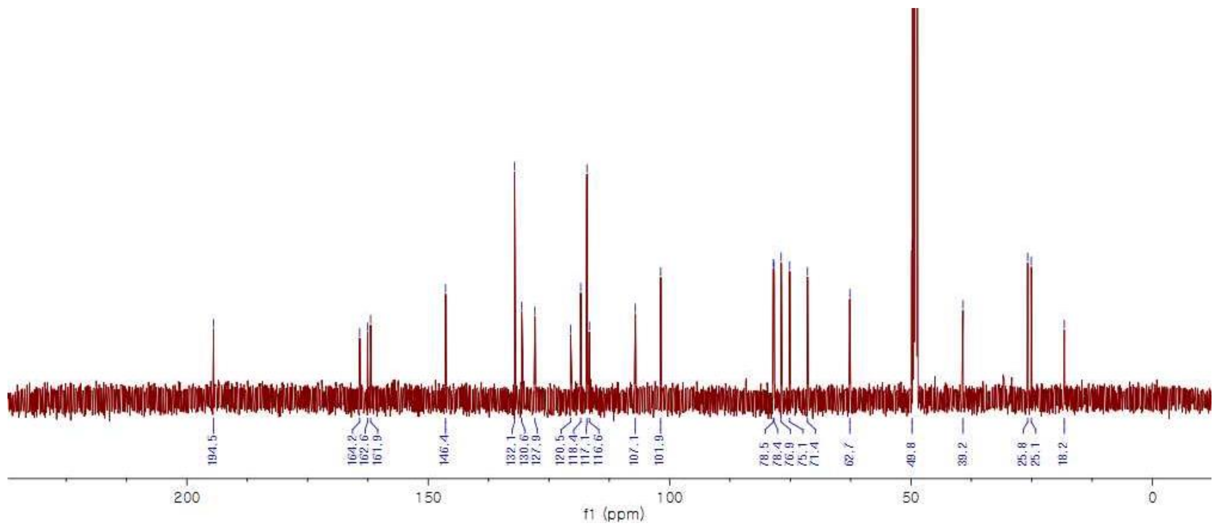

Figure S15. ¹³C-NMR (150 MHz, methanol-d_4) spectrum of **5**

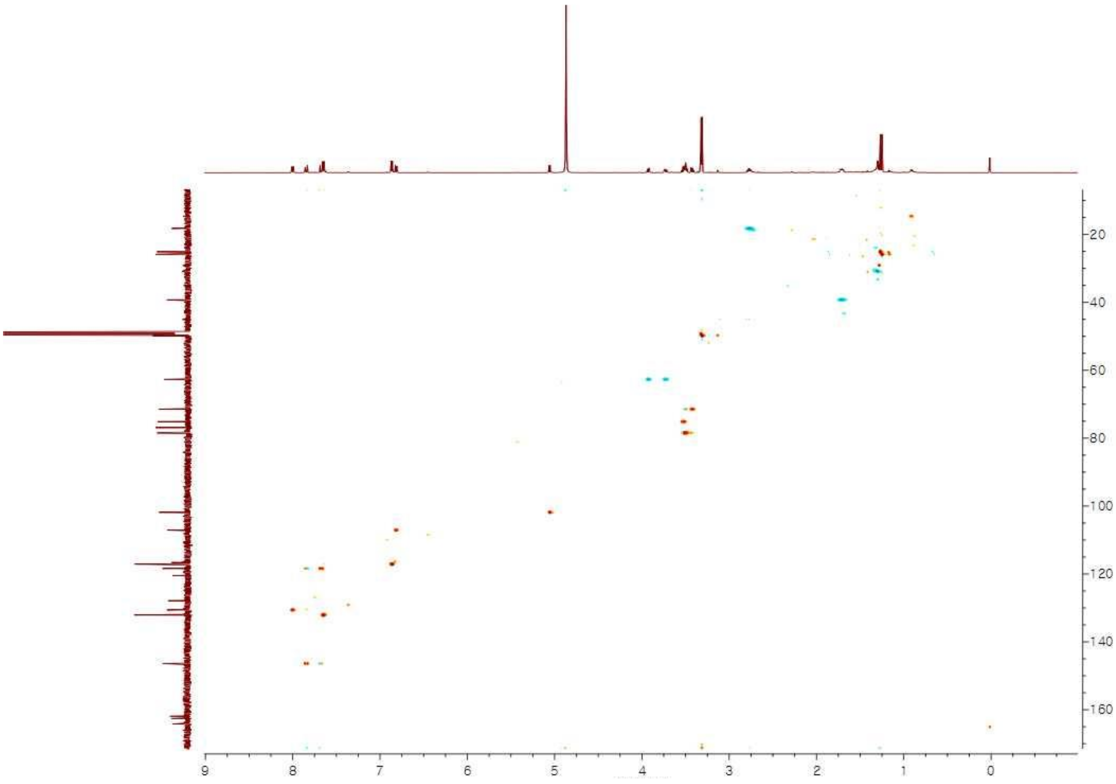

Figure S16. HSQC spectrum of **5**

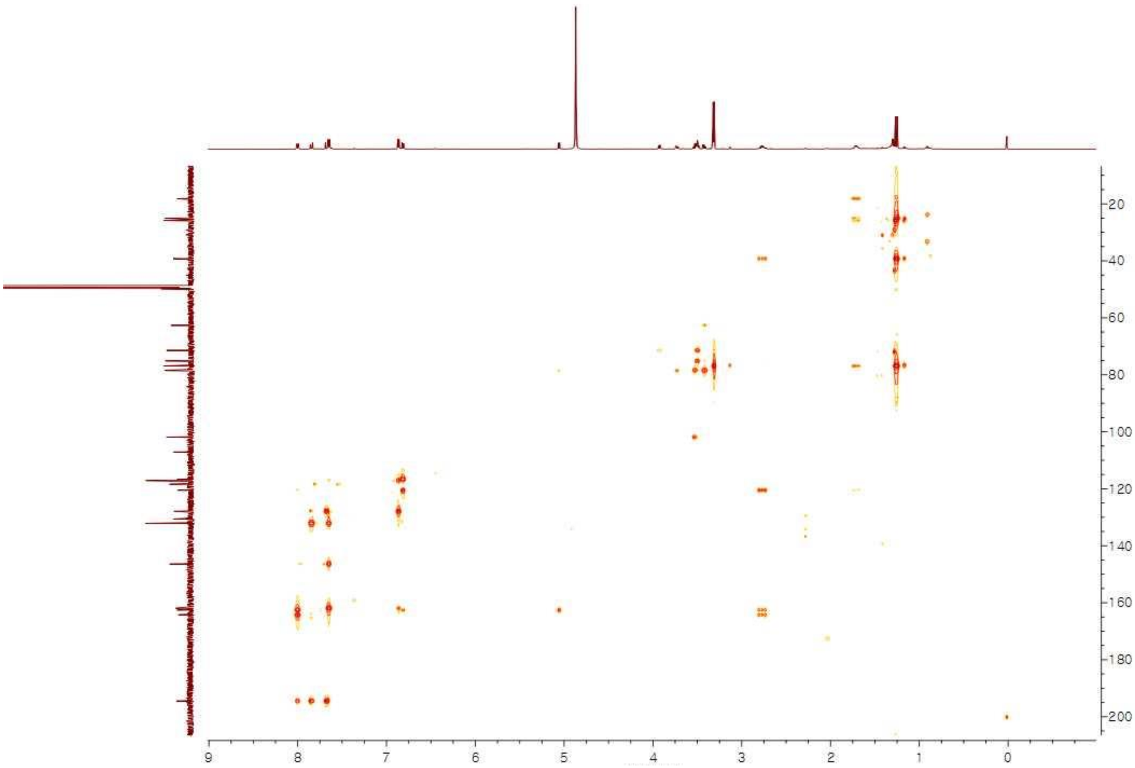

Figure S17. HMBC spectrum of **5**

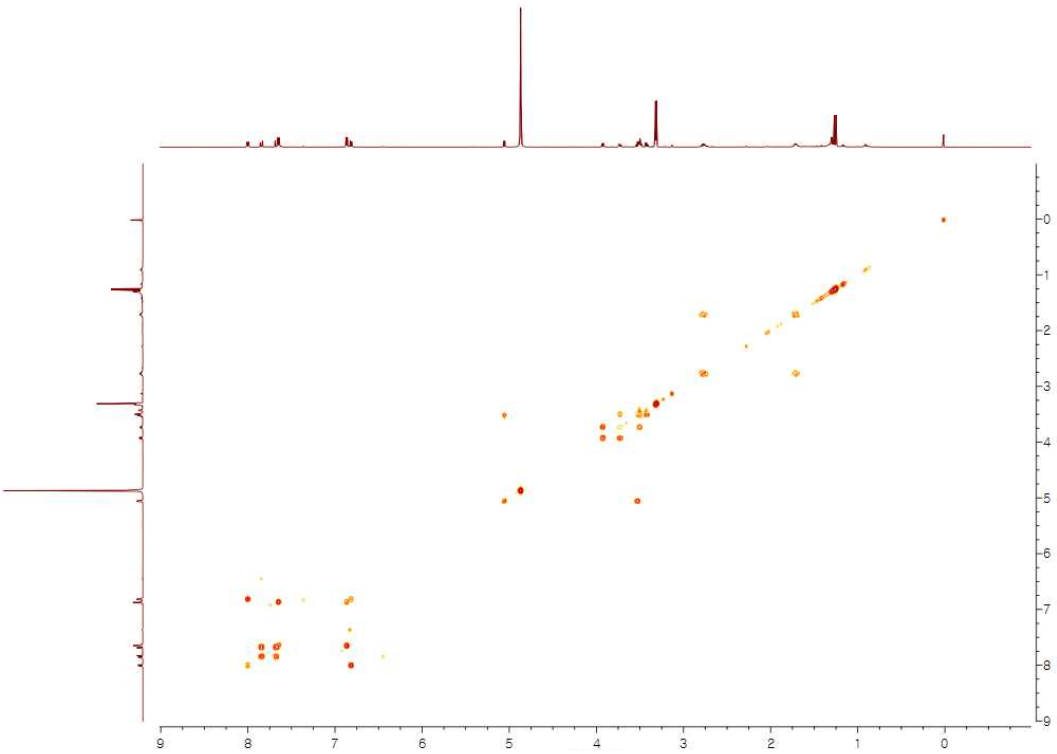

Figure S18. COSY spectrum of **5**

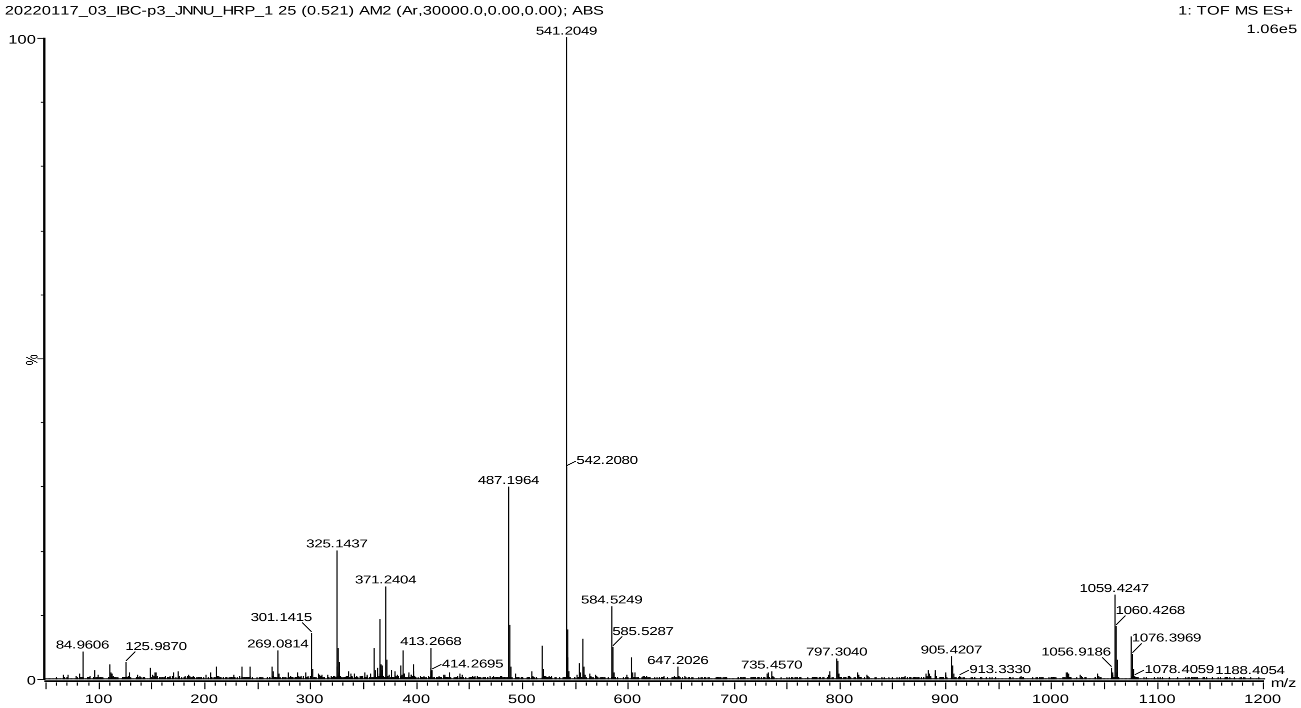

Elemental Composition Report

Single Mass Analysis
Tolerance = 5.0 PPM / DBE: min = -3.0, max = 200.0
Element prediction: Off
Number of isotope peaks used for i-FIT = 3

Monoisotopic Mass, Even Electron Ions
Elements Used:
C: 0-35 H: 0 -50 O: 0 -10 Na: 0 -1

Minimum:				-3.0				
Maximum:		300.0	5.0	200.0				
Mass	Calc. Mass	mDa	PPM	DBE	i-FIT	Norm	Conf(%)	Formula
519.2230	519.2230	0.0	0.0	10.5	397.1	0.158	85.36	C27 H35 O10
	519.2206	2.4	4.6	7.5	398.9	1.921	14.64	C25 H36 O10 Na
541.2049	541.2050	-0.1	-0.2	10.5	590.6	1.548	21.28	C27 H34 O10 Na
	541.2074	-2.5	-4.6	13.5	589.3	0.239	78.72	C29 H33 O10

Figure S19. HRESIMS spectrum of **5**

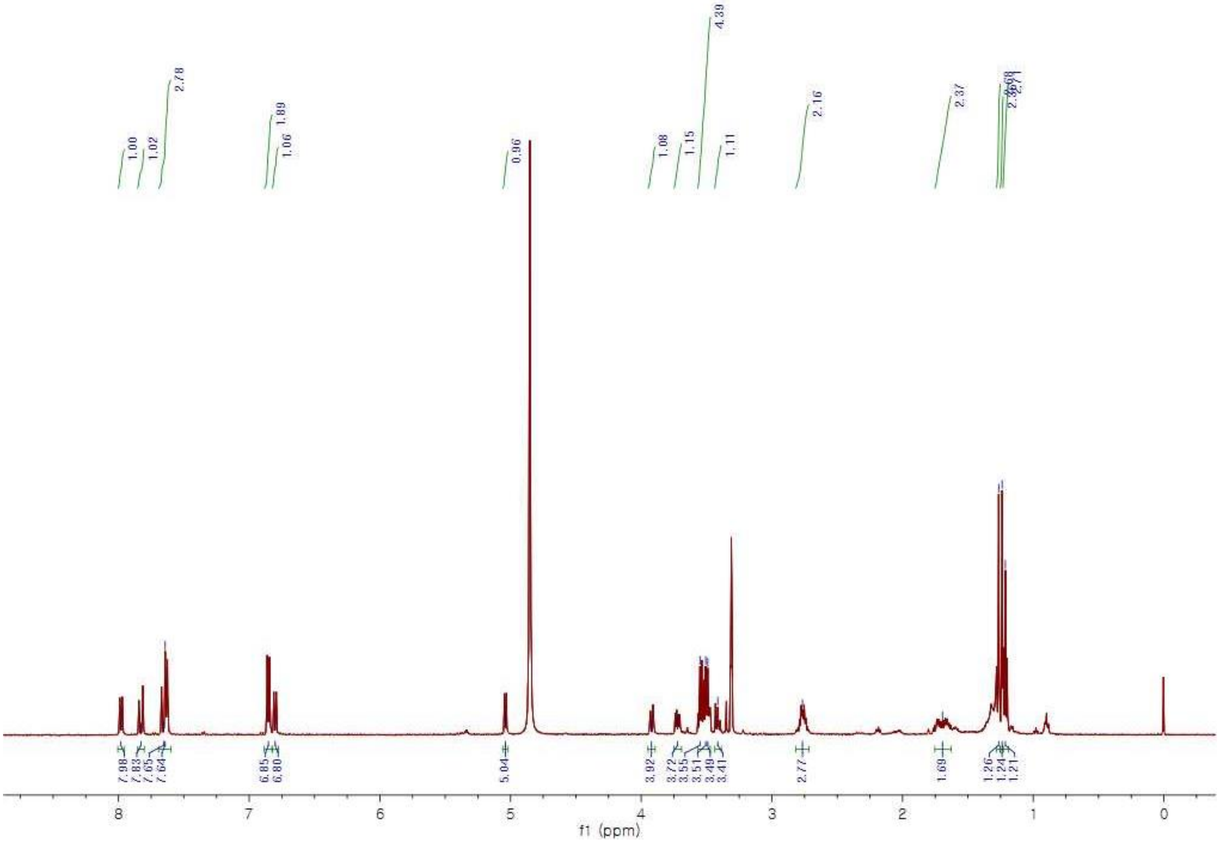

Figure S20. ^{1}H-NMR (500 MHz, methanol-d_4) spectrum of **7**

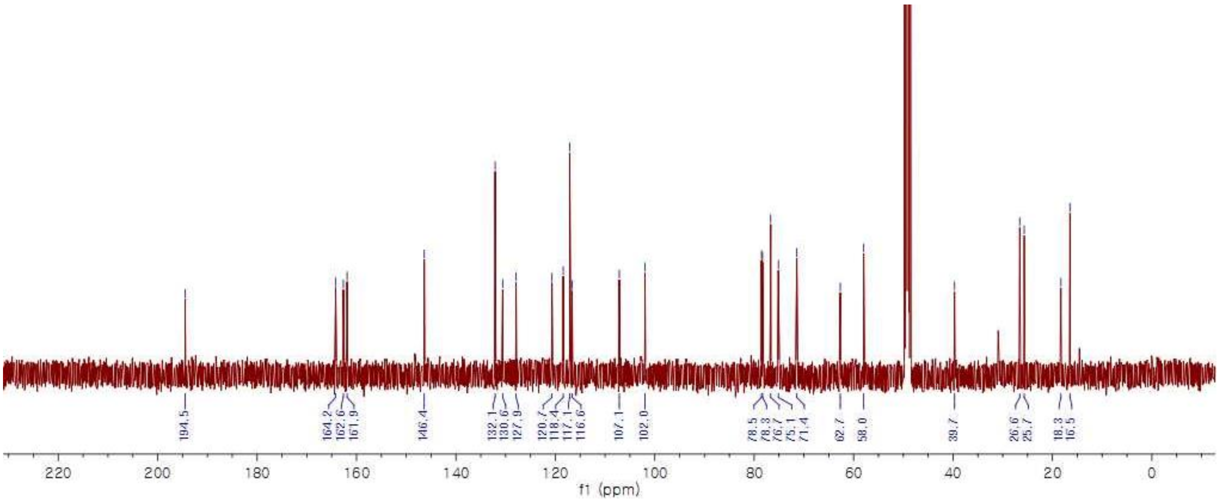

Figure S21. ^{13}C-NMR (125 MHz, methanol-d_4) spectrum of **7**

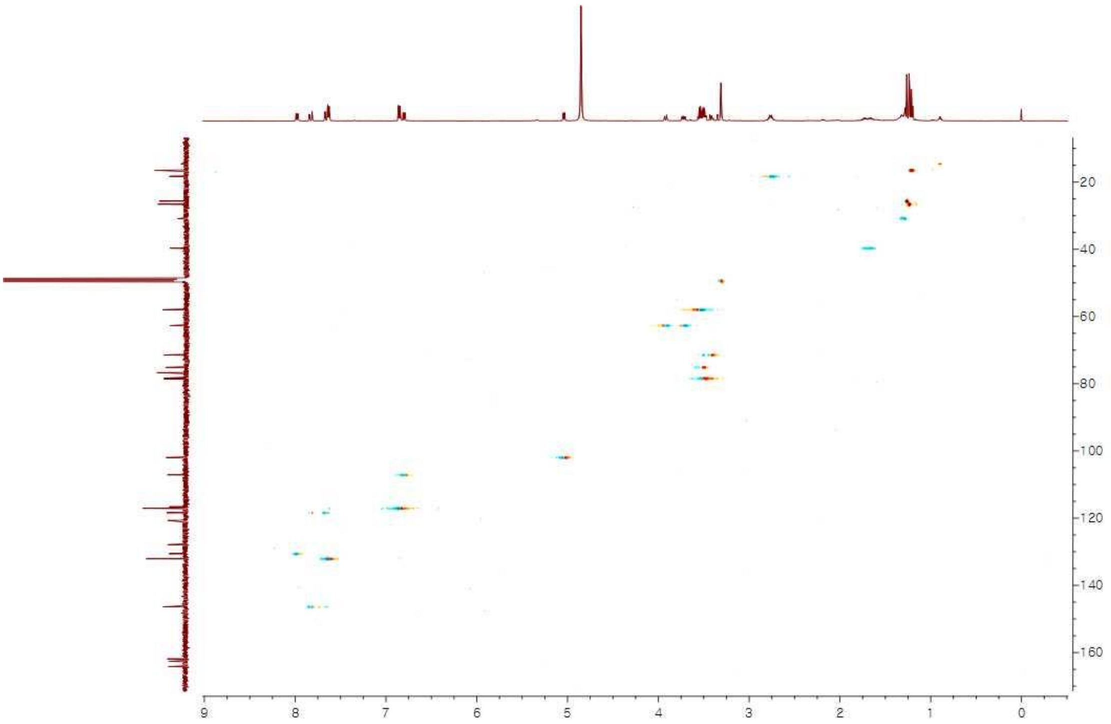

Figure S22. HSQC spectrum of **7**

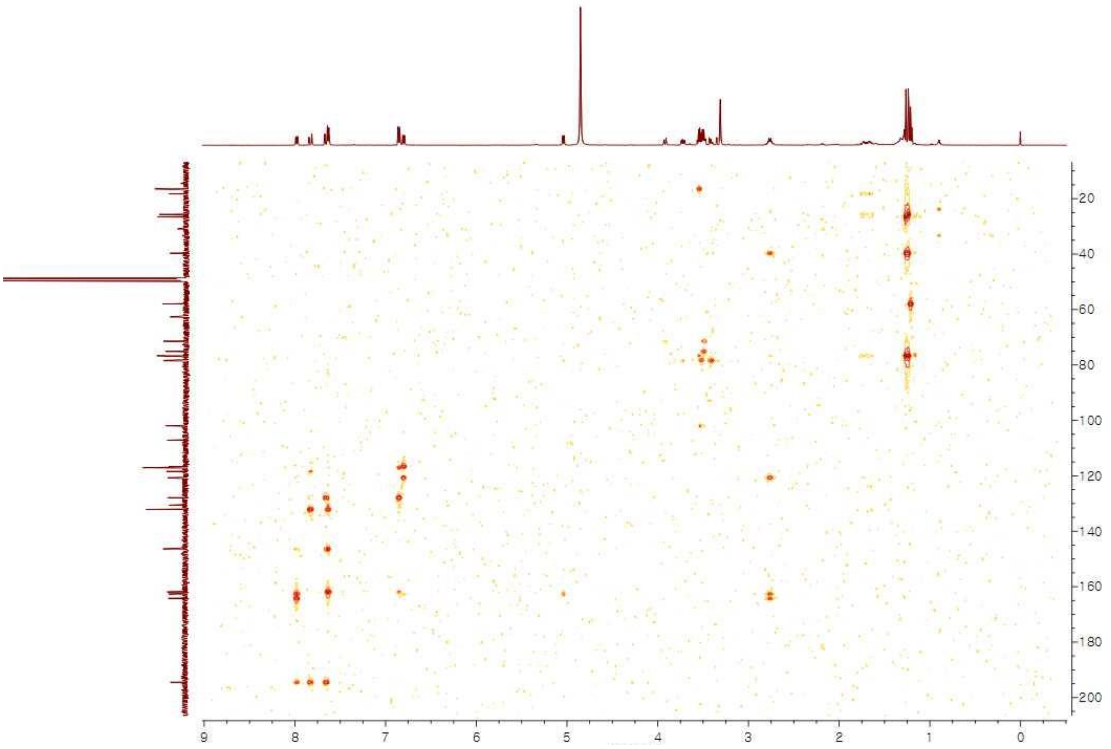

Figure S23. HMBC spectrum of **7**

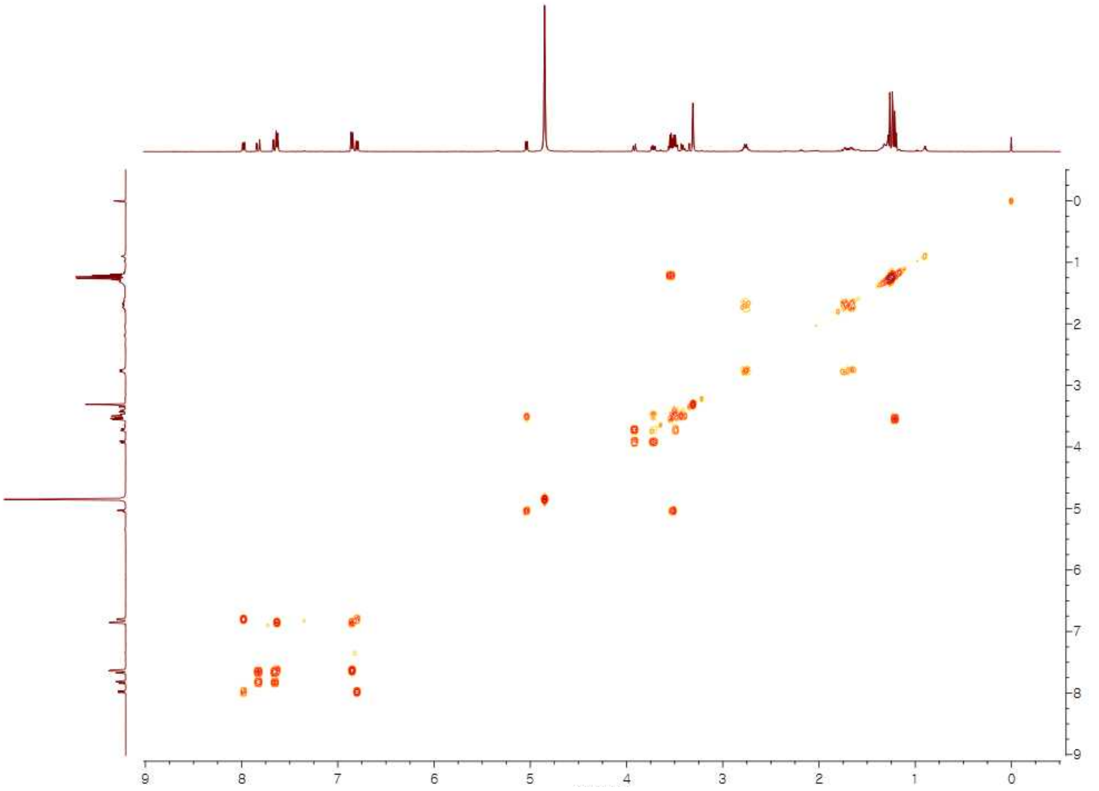

Figure S24. COSY spectrum of **7**

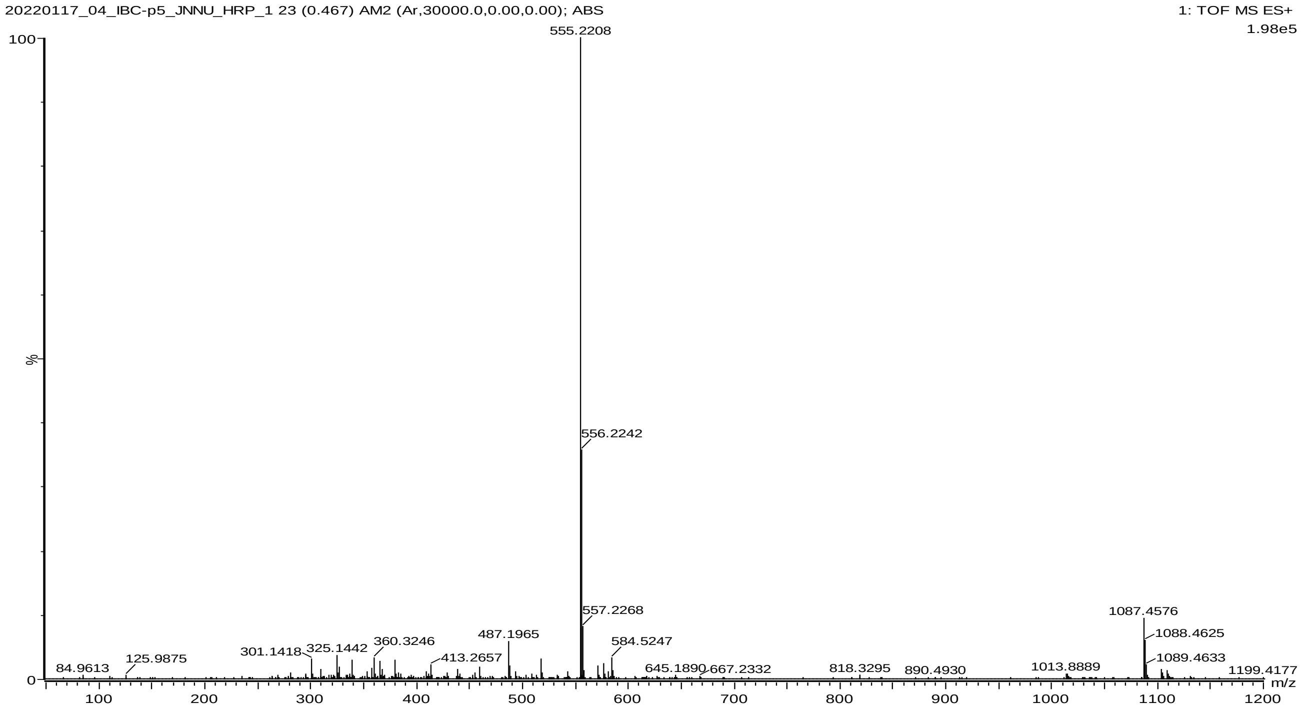

Elemental Composition Report

Single Mass Analysis
Tolerance = 5.0 PPM / DBE: min = -3.0, max = 200.0
Element prediction: Off
Number of isotope peaks used for i-FIT = 3

Monoisotopic Mass, Even Electron Ions
47 formula(e) evaluated with 2 results within limits (all results (up to 1000) for each mass)
Elements Used:
C: 0-35 H: 0 -50 O: 0 -10 Na: 0 -1

Minimum:				-3.0				
Maximum:		300.0	5.0	200.0				
Mass	Calc. Mass	mDa	PPM	DBE	i-FIT	Norm	Conf(%)	Formula
555.2208	555.2206	0.2	0.4	10.5	663.1	0.784	45.65	C28 H36 O10 Na
	555.2230	-2.2	-4.0	13.5	662.9	0.610	54.35	C30 H35 O10

Figure S25. HRESIMS spectrum of **7**

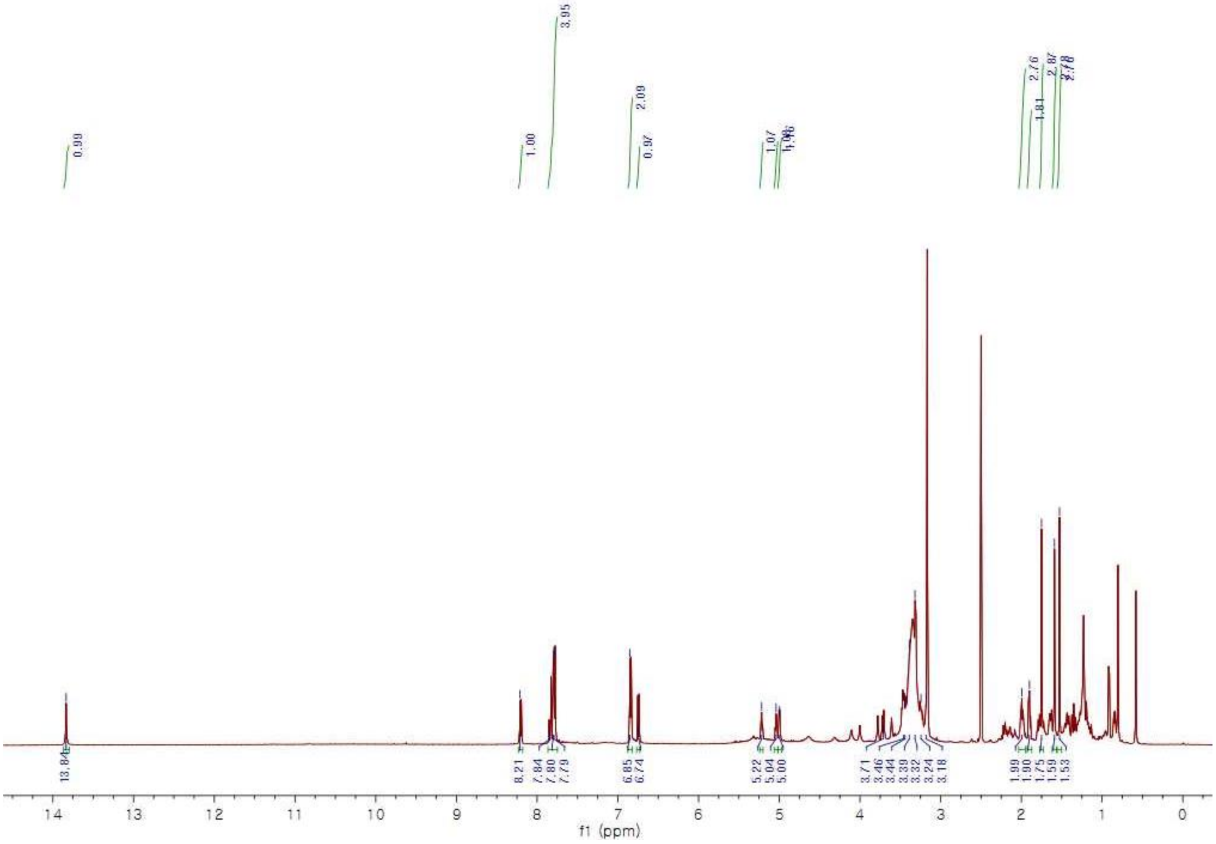

Figure S26. ^{1}H-NMR (600 MHz, DMSO-d_6) spectrum of **10**

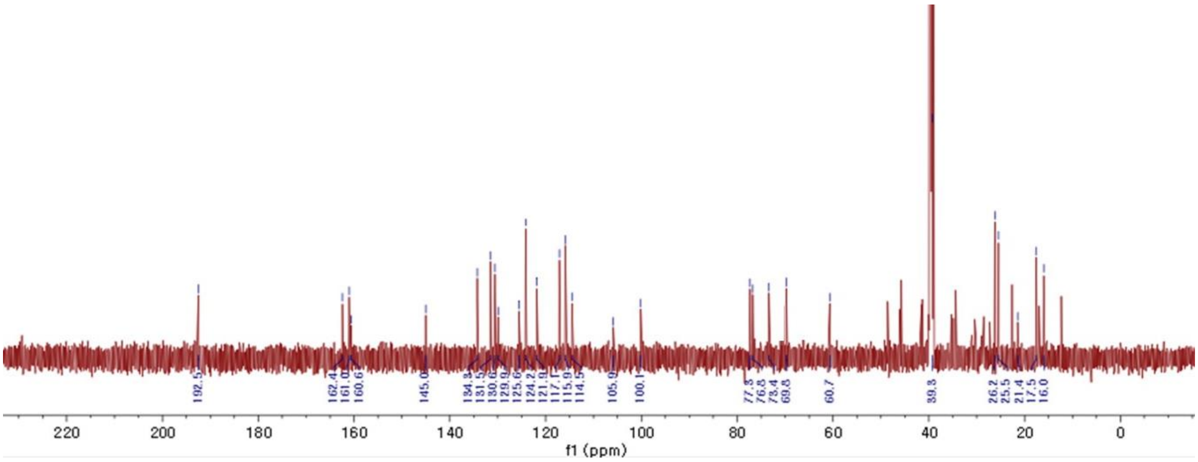

Figure S27. ^{13}C-NMR (150 MHz, DMSO-d_6) spectrum of **10**

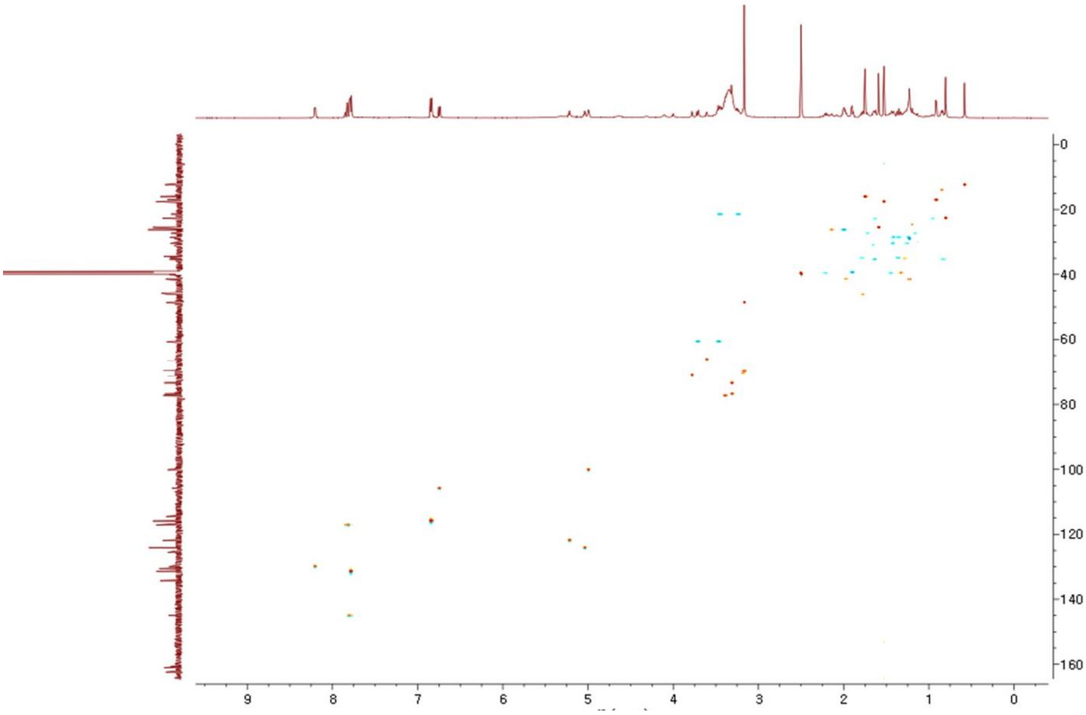

Figure S28. HSQC spectrum of **10**

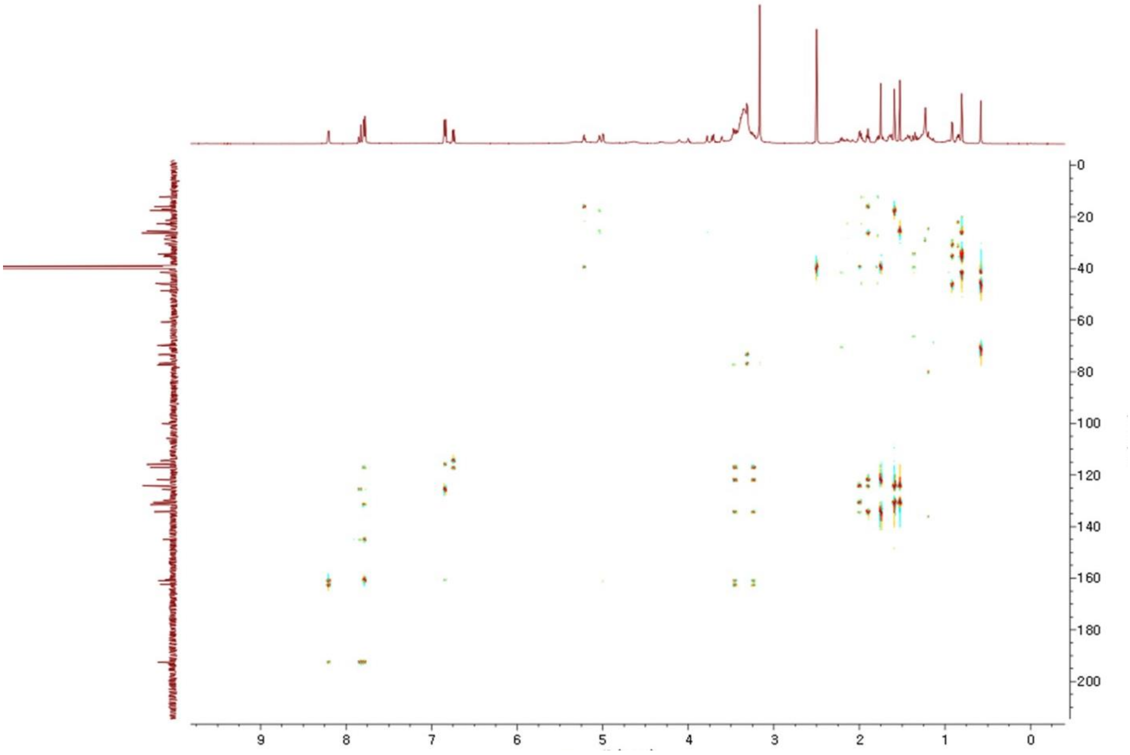

Figure S29. HMBC spectrum of **10**

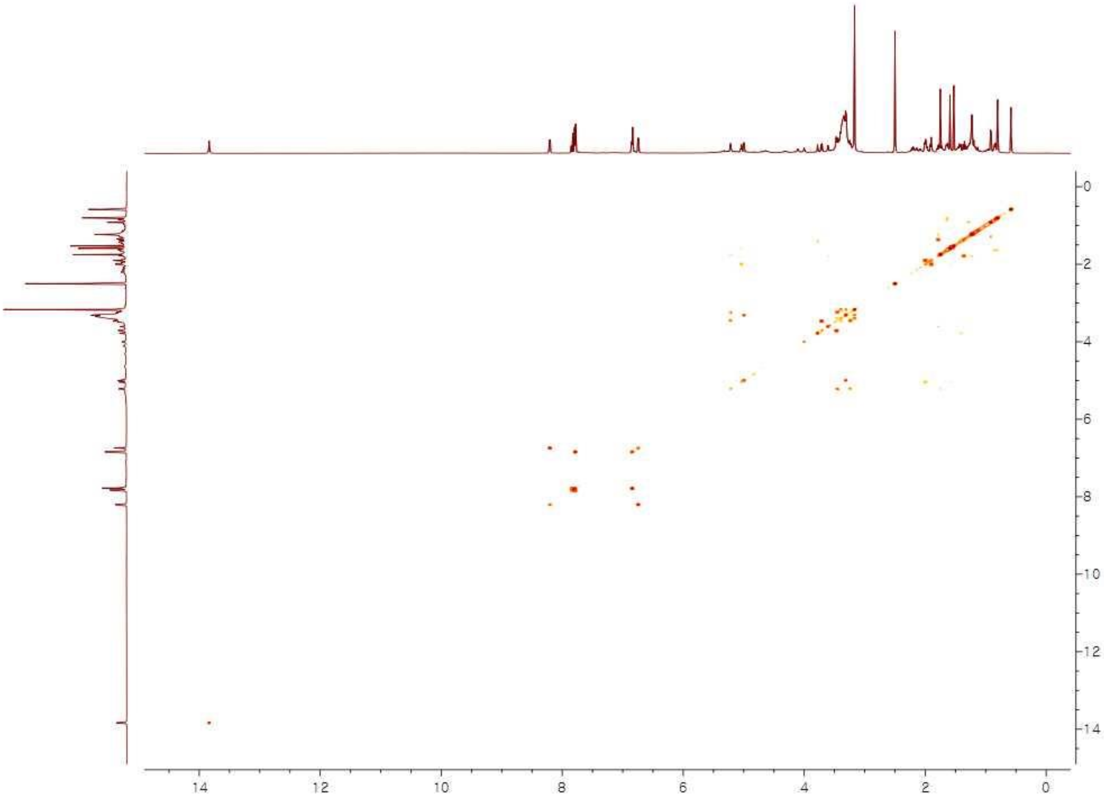

Figure S30. COSY spectrum of **10**

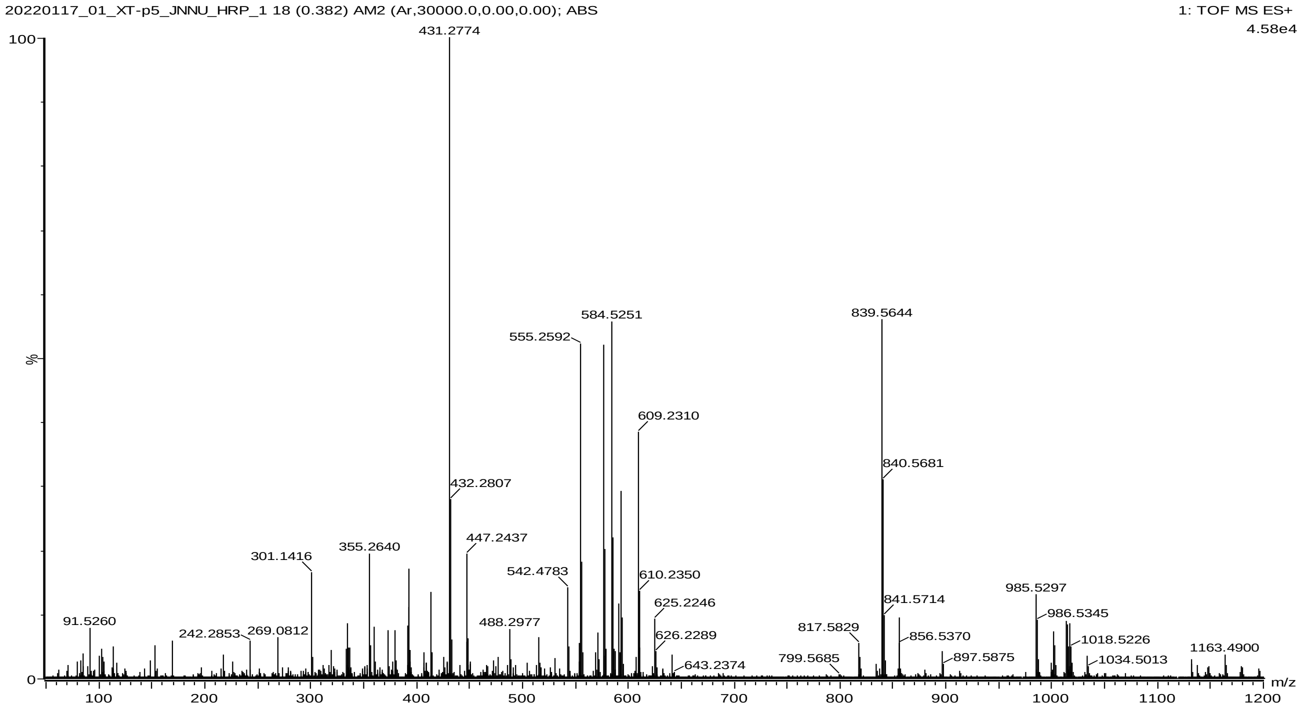

Elemental Composition Report

Single Mass Analysis
Tolerance = 5.0 PPM / DBE: min = -3.0, max = 200.0
Element prediction: Off
Number of isotope peaks used for i-FIT = 3

Monoisotopic Mass, Even Electron Ions
Elements Used:
C: 0-35 H: 0 -50 O: 0 -10 Na: 0 -1

Minimum:				-3.0				
Maximum:		300.0	5.0	200.0				
Mass	Calc. Mass	mDa	PPM	DBE	i-FIT	Norm	Conf(%)	Formula
555.2592	555.2594	-0.2	-0.4	12.5	488.7	0.000	99.96	C31 H39 O9
	555.2570	2.2	4.0	9.5	496.4	7.719	0.04	C29 H40 O9 Na
577.2414	577.2414	0.0	0.0	12.5	438.4	1.677	18.70	C31 H38 O9 Na
	577.2438	-2.4	-4.2	15.5	437.0	0.207	81.30	C33 H37 O9

Figure S31. HRESIMS spectrum of **10**

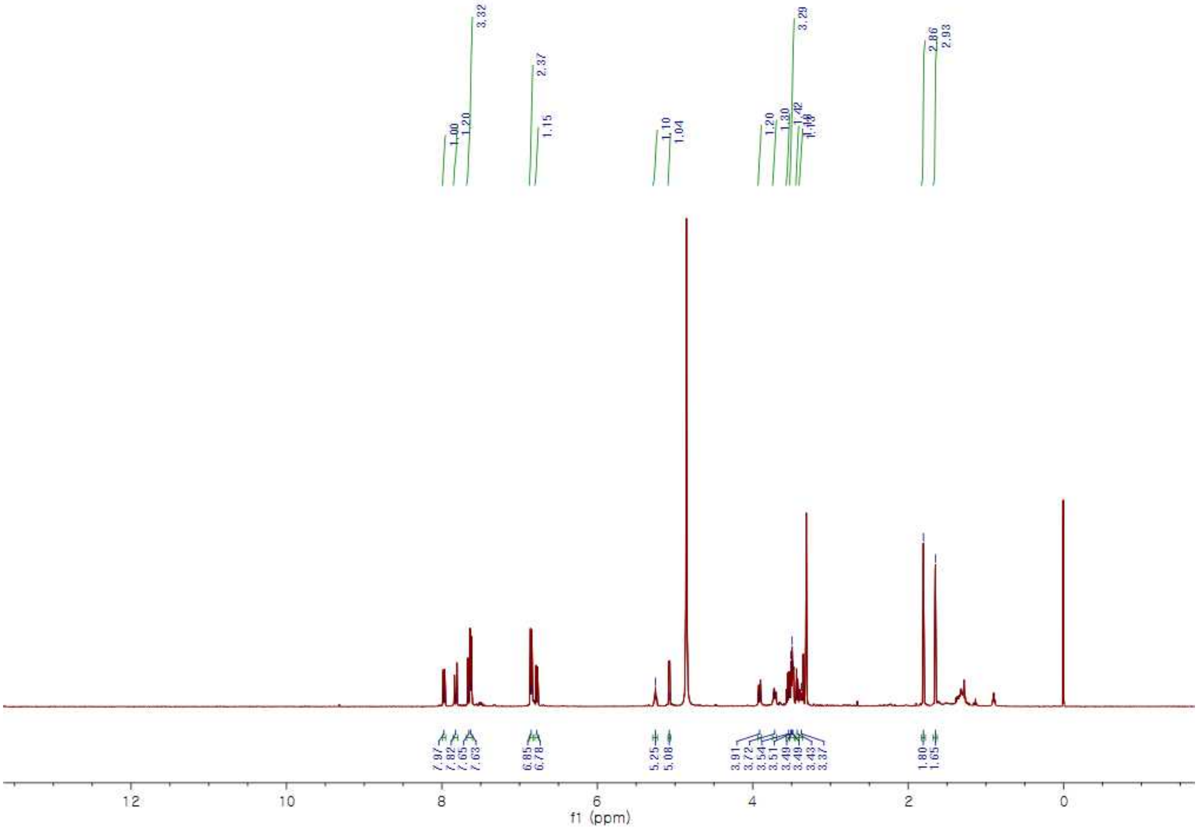

Figure S32. ^{1}H-NMR (500 MHz, methanol-d_4) spectrum of **6**

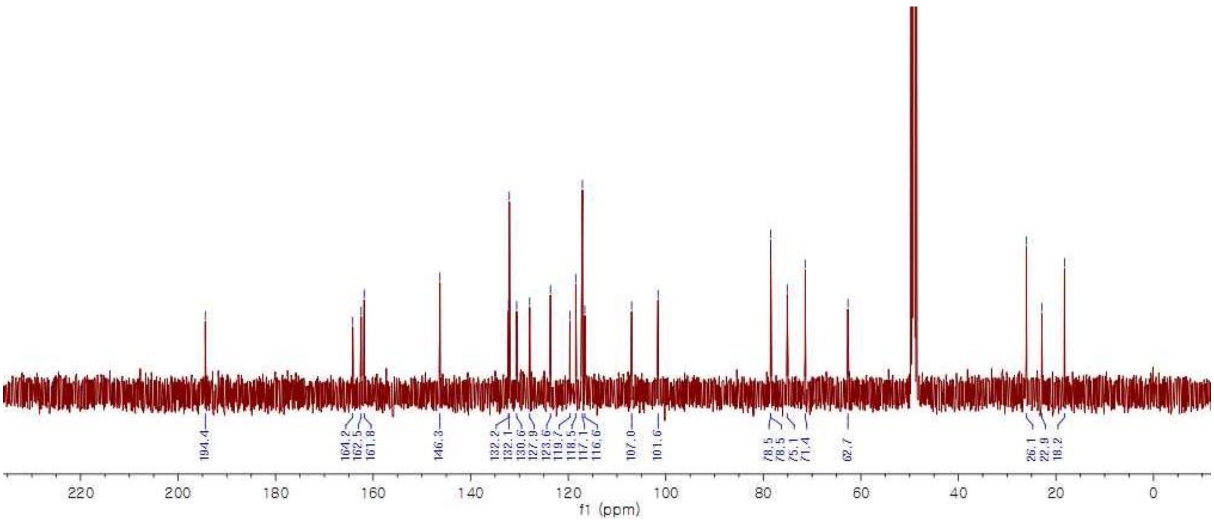

Figure S33. ^{13}C-NMR (125 MHz, methanol-d_4) spectrum of **6**

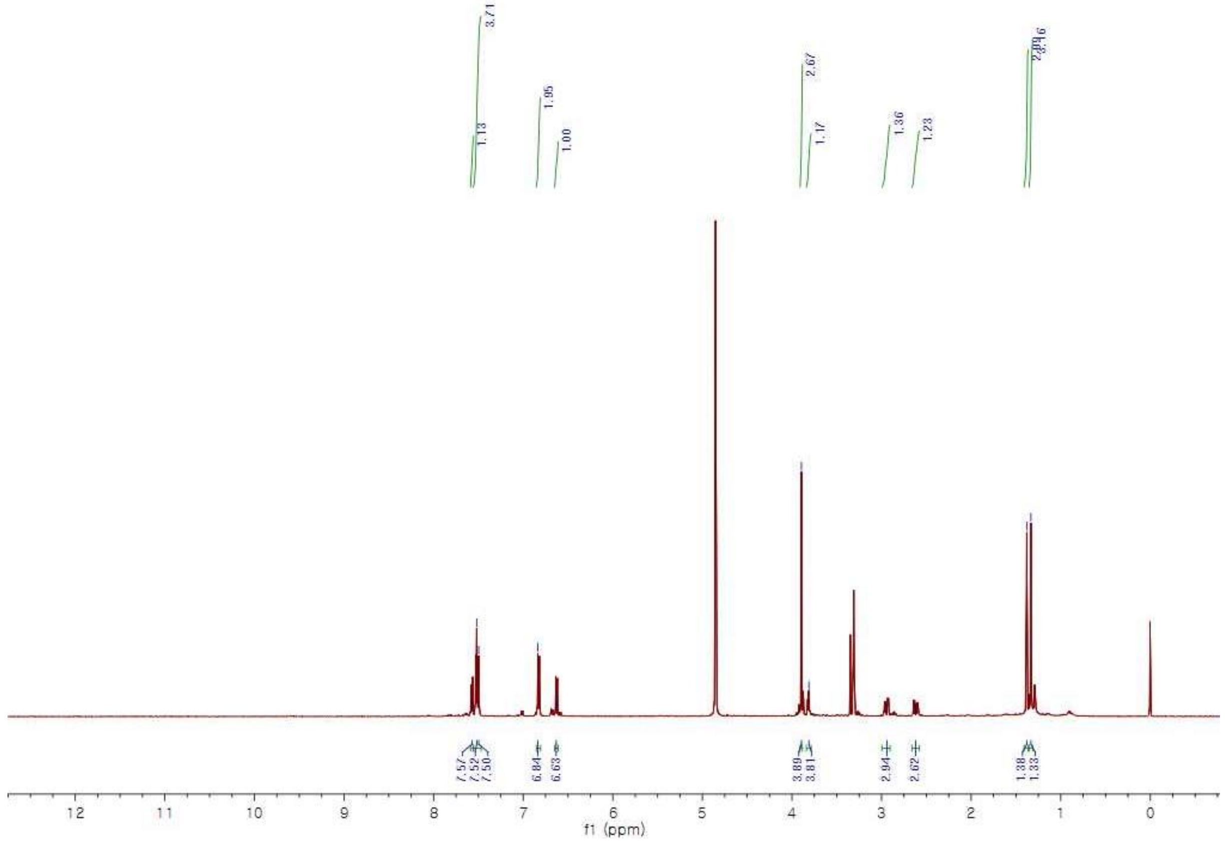

Figure S34. ^{1}H-NMR (500 MHz, methanol-d_4) spectrum of **8**

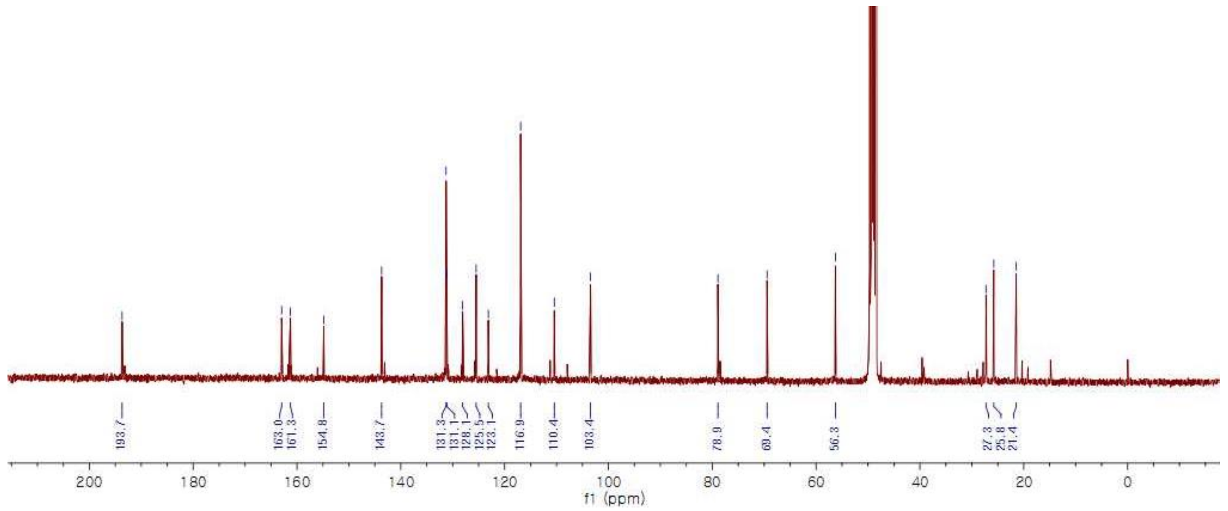

Figure S35. ^{13}C-NMR (100 MHz, methanol-d_4) spectrum of **8**

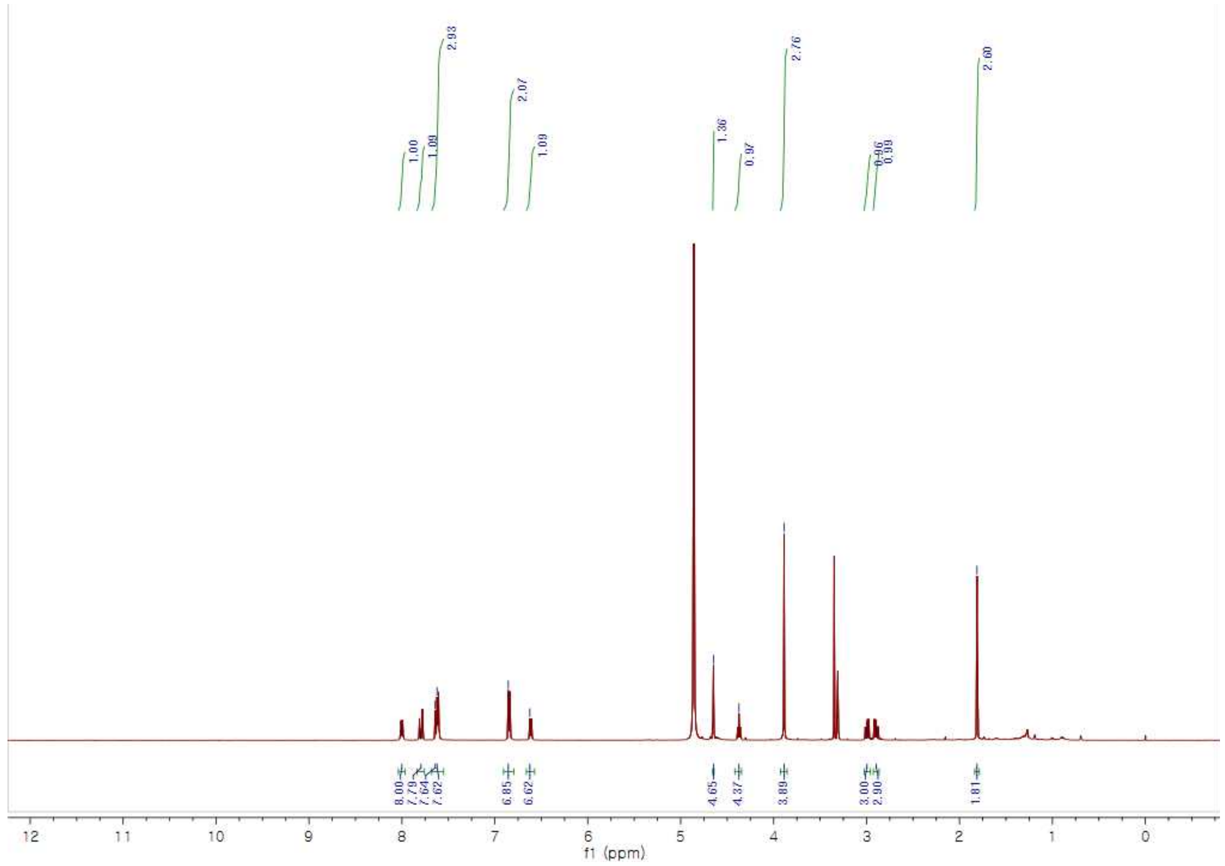

Figure S36. ^{1}H-NMR (500 MHz, methanol-d_4) spectrum of **9**

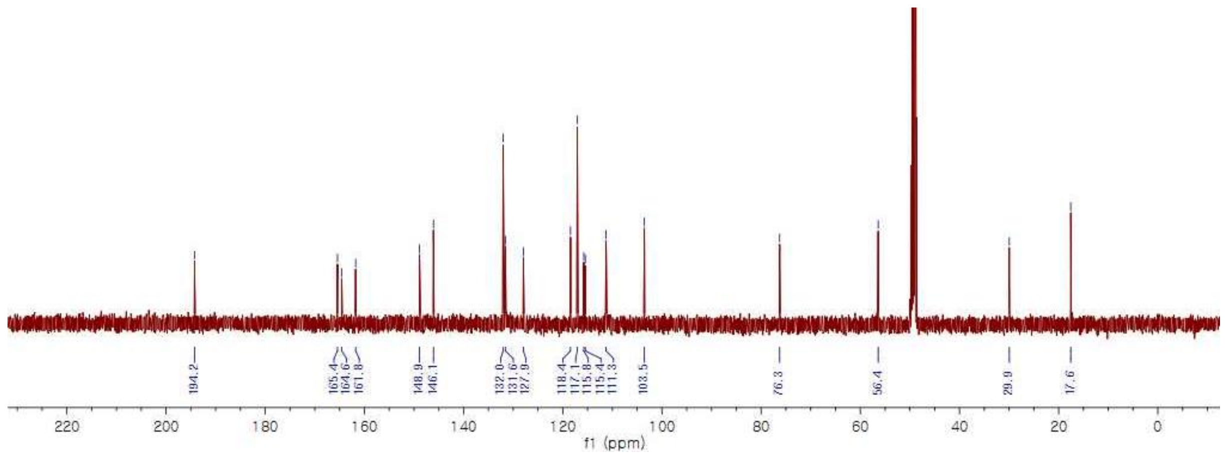

Figure S37. ^{13}C-NMR (125 MHz, methanol-d_4) spectrum of **9**

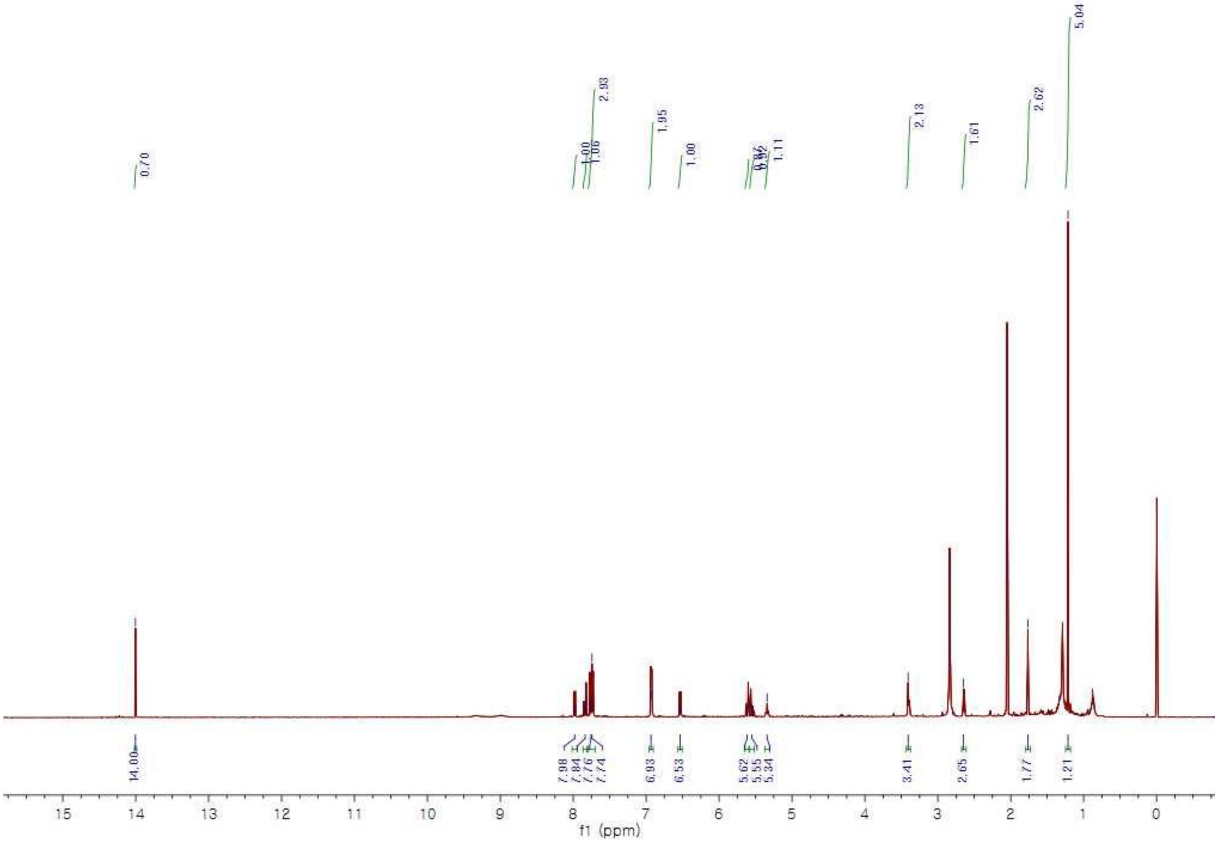

Figure S38. ^{1}H-NMR (500 MHz, acetone-d_6) spectrum of **11**

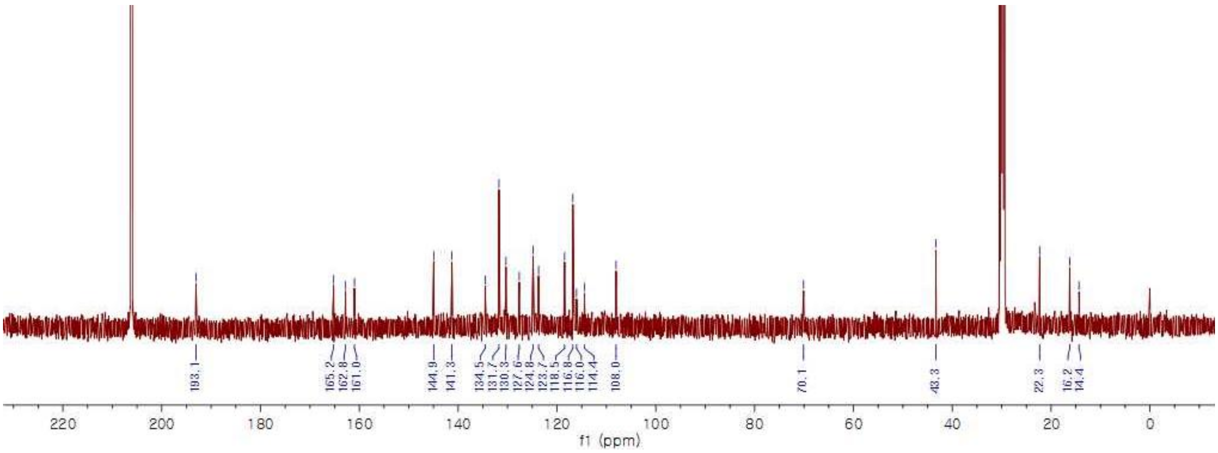

Figure S39. ^{13}C-NMR (125 MHz, acetone-d_6) spectrum of **11**

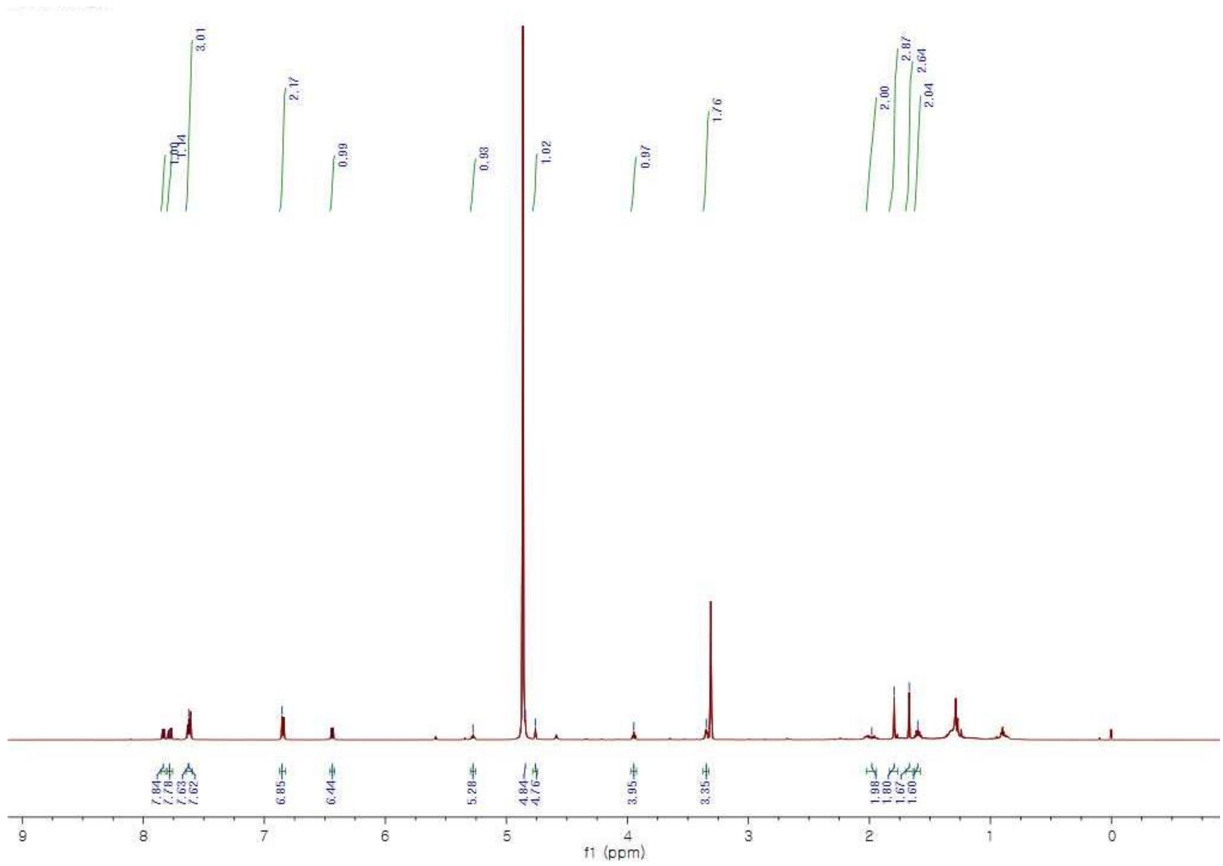

Figure S40. ^{1}H-NMR (600 MHz, methanol-d_4) spectrum of **12**

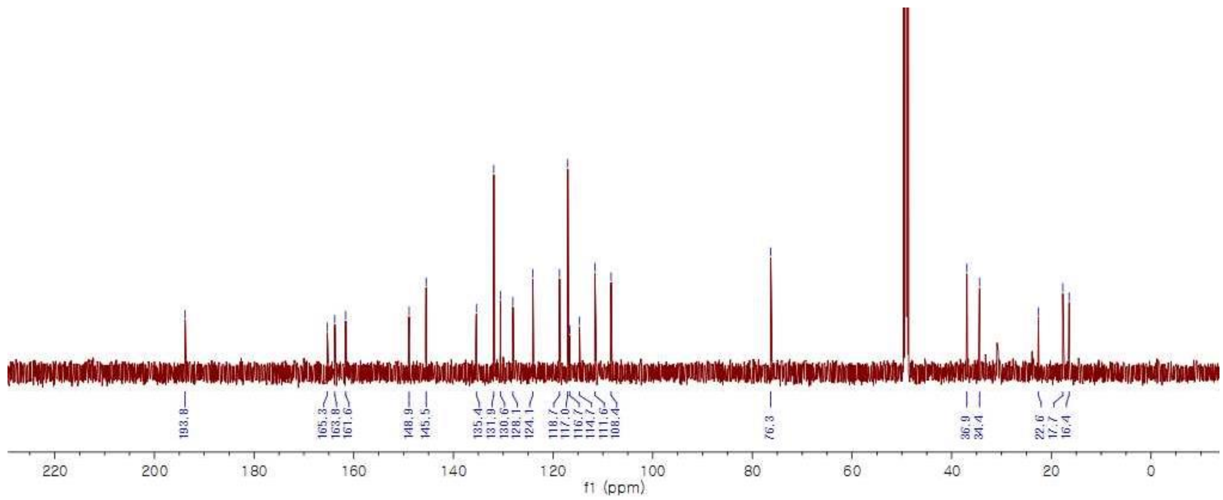

Figure S41. ^{13}C-NMR (150 MHz, methanol-d_4) spectrum of **12**
